# Acoustic Rising Microbubbles for Efficient Liquid Operations

**DOI:** 10.34133/cbsystems.0449

**Published:** 2026-03-09

**Authors:** Chenhao Bai, Zhuo Chen, Yunsheng Li, Yan Chen, Qing Shi, Qiang Huang, Toshio Fukuda, Tatsuo Arai, Xiaoming Liu

**Affiliations:** ^1^ School of Mechatronics Engineering and Key Laboratory of Biomimetic Robots and Systems, Ministry of Education, Beijing Institute of Technology, Beijing 100081, China.; ^2^ Thrust of Robotics and Autonomous Systems, The Hong Kong University of Science and Technology (Guangzhou), Guangzhou 511453, China.; ^3^ Institute of Innovation for Future Society, Nagoya University, Nagoya 4648601, Japan.; ^4^ Center for Neuroscience and Biomedical Engineering, The University of Electro-Communications, Tokyo 1828585, Japan.; ^5^ School of Medical Engineering, Beijing Institute of Technology, Zhuhai 519088, China.

## Abstract

Efficient liquid manipulation is crucial in chemical engineering, biological research, clinical applications, and materials science. Bubbles, such as boiling, rising, and cavitating bubbles, have been widely employed to enhance mixing and mass transfer through their unique hydrodynamic behaviors. Yet, conventional bubble-based approaches often face limited scalability and poor performance in high-viscosity environments. Here, we introduce a strategy that employs low-energy acoustic excitation of rising microbubbles to achieve scalable and efficient mass transfer across macroscale and microscale domains. By coupling buoyancy-driven convection with localized acoustic microstreaming, acoustic rising microbubbles simultaneously extend the operational workspace and intensify local mass transfer. Particle image velocimetry and computational fluid dynamics analyses characterize the distinct contributions of buoyancy-induced flows, acoustically induced microstreaming, and their superimposed effects. Various chemical and biomedical applications, including efficient high-viscosity mixing, accelerated chemical material synthesis, altered cell membrane permeability, promoted cell lysis, and thrombus clearance, demonstrate the great potential of the proposed acoustic rising bubbles for efficient mass transfer in laboratory and industrial liquid manipulations.

## Introduction

Efficient liquid manipulation is central to chemical engineering, biological research, clinical practice, and materials science, where processes such as mixing, mass transfer, and reaction control dictate performance and scalability [1–3]. Among various strategies, bubbles have long been exploited due to their unique dynamics. The formation, growth, and collapse of bubbles create complex multiphase flows that enhance local transport processes [4–6]. In heat exchangers and gas–liquid reactors, for instance, boiling bubbles accelerate heat transfer and chemical reactions [7–9]. Similarly, rising bubbles driven by buoyancy generate large-scale circulation, improving gas absorption and fluid homogenization in reactors and material fabrication systems [10–13]. Cavitation bubbles have also been widely investigated in biomedical contexts, enabling targeted drug delivery and tissue penetration [14–16]. Despite this versatility, conventional bubble-based systems face critical limitations in scalability and efficiency when tasked with homogenizing high-viscosity liquids or achieving uniform mass transfer across both macroscopic and microscopic length scales.

Macroscopic mixing techniques such as mechanical stirring and bubble column reactors are effective for generating bulk agitation, turbulence, and enhanced gas–liquid exchange and are indispensable in large-scale applications ranging from wastewater treatment to chemical synthesis [17–19]. However, their effectiveness diminishes at low Reynolds numbers, as viscous damping suppresses turbulent cascades and prevents efficient microscale mass transfer [20–22]. Conversely, microfluidic technologies have enabled elegant control of mixing under laminar conditions through channel designs and passive micromixers [23], such as herringbone or zigzag patterns, which promote chaotic advection and interfacial stretching [24,25]. While highly efficient in small volumes [26], these approaches suffer from low throughput and remain difficult to scale beyond laboratory applications [27].

At the microscopic scale, microfluidic devices have revolutionized liquid operations at low Reynolds numbers. By incorporating sharp-edged features [28–30], researchers induced chaotic advection and enhanced interfacial mass transfer for efficient mixing operation at the microscale [31–34]. These passive micromixers excel in small-volume assays, and their inherently laminar flows and limited throughput hinder scaling up to industrial demands [35–37]. Increasing flow rates offer only marginal gains in mixing volume [38], meaning most designs remain confined to laboratory applications such as diagnostics and chemical assays featuring small-volume liquid samples [39–41]. Acoustic waves provide a compelling strategy to overcome these limitations by introducing local microstreaming that disrupts laminar boundaries and accelerates convective transport [42–46]. When acoustic fields excite the oscillation of structures or multiphase systems [47–49], they generate vortical microflows that substantially enhance diffusion and mass transfer, improving reaction kinetics and homogenization in microscale environments [1,50,51]. Despite these advantages, acoustofluidic mixing remains largely disconnected from bulk processes and often requires cooperation with microfluidic chips. Recently, pioneering research realized programmable acoustic mixing in a large open workspace by mobilizing the acoustically driven structures and multiphase system, inspiring us to move acoustic microbubbles together with the microstreaming in a large space for scaling up the conventional acoustically driven liquid operations [52–54].

**Fig. 1.**
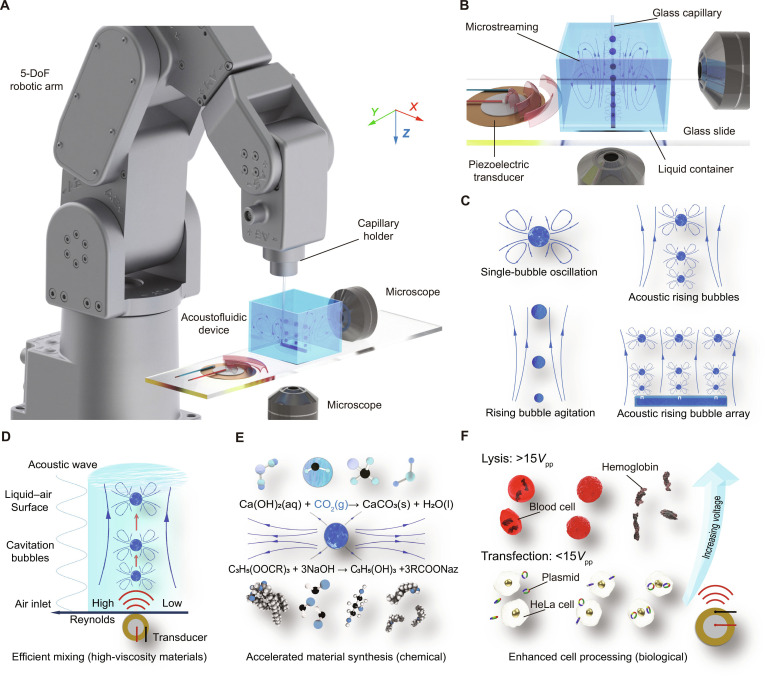
Acoustic rising bubbles generate strong shear flows and enable rapid mixing and cell manipulation. (A) Setup comprising a robotic arm, a glass capillary, a syringe pump, and an acoustofluidic device with integrated imaging. (B) Large-scale shear flow produced by rising bubbles. (C) Schematics of different flow modes: single oscillation, buoyancy-driven rise, acoustic rising, and bubble array mixing. (D) Enhanced mass transfer through disrupted laminar flow. (E) Accelerated chemical reactions by bubble microstreaming. (F) Shear-induced cell membrane permeabilization and lysis. DoF, degree of freedom.

Here, we demonstrate that rising bubbles subjected to low-frequency acoustic fields can simultaneously drive buoyancy-induced bulk flows and localized acoustic microstreaming (Fig. 1A). Unlike traditional bubble columns that rely solely on buoyancy, these acoustic rising bubbles generate coupled large-scale agitation and microscale vortices during their ascent (Fig. 1B), substantially enhancing mixing efficiency even in viscous fluids. By arranging bubbles in arrays, the influence region can be expanded, enabling high-throughput operations without complex mechanical stirring or microchannel confinement (Fig. 1C). We further show that this approach accelerates chemical material synthesis and supports biomedical processes such as membrane permeabilization, thrombus clearance, and red blood cell lysis, thereby highlighting its versatility and scalability (Fig. 1D to F). This dual action of buoyancy-driven transport and acoustically induced microstreaming establishes acoustic rising bubbles as a promising platform for liquid operations across chemical, biological, and industrial contexts.

## Methods

### Acoustic rising microbubbles

Acoustically driven bubble oscillations have been utilized to enhance mass transfer efficiency in microchannels, while rising bubbles are widely employed for large-scale mixing in industrial bubble column reactors [4,46]. Here, we introduce the concept of tunable, acoustically actuated rising microbubbles, which combine large-scale, buoyancy-driven convection from the rising bubble with localized microstreaming effects generated by the bubble in an acoustic field. This synergy achieves efficient mass transfer and mixing across both macroscopic and microscopic scales.

**Fig. 2.**
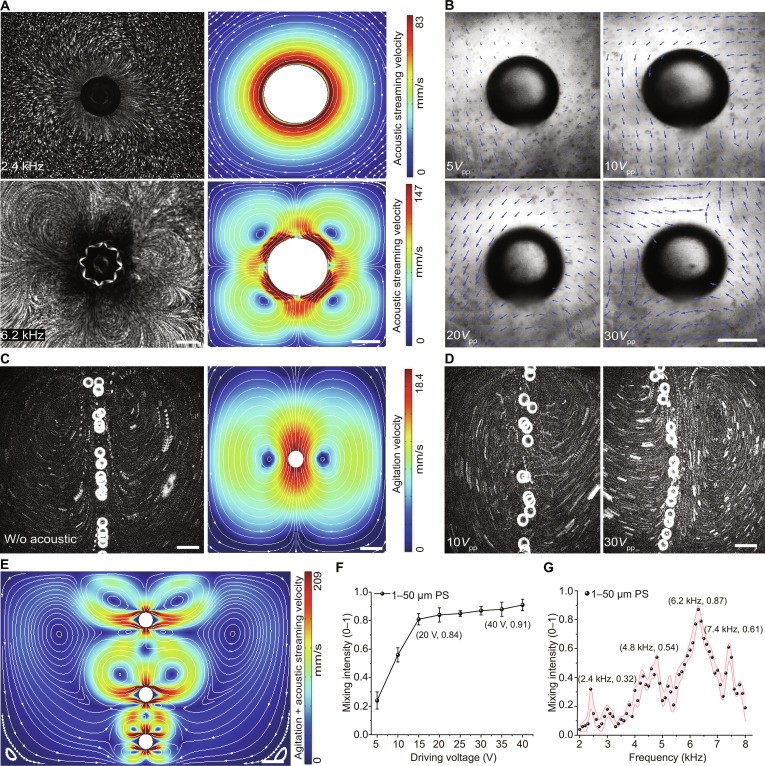
Acoustic rising bubbles disrupt laminar boundaries and achieve rapid, large-scale mixing. (A) Experiments and simulations showing resonance-induced mixing enhancement. (B) Side view of a suspended bubble at the resonance frequency with increasing voltage. (C) Buoyancy-driven flow from rising bubbles without acoustics. (D) Acoustic rising bubbles produce fine and extensive mixing at the resonance frequency. (E) Simulation of large-scale rapid mixing around acoustic bubbles. (F) Effect of voltage on mixing intensity of acoustic rising bubbles. (G) Effect of frequency on mixing intensity at 15*V*_pp_. Scale bars: 200 μm in (A) and (B); 1 mm in (C) to (E). Shaded error bands represent the standard deviation (*n* = 3). PS, polystyrene.

We characterized the bubble dynamics through micro-particle image velocimetry experiments (Figs. S1 to S8) and computational fluid dynamics simulations (Figs. S9 to S12). Numerical results (Sections S1 to S3) demonstrate that when a microbubble is acoustically excited at its resonance frequency (6.2 kHz), it generates high-velocity streaming vortices in its vicinity, notably enhancing mass transfer efficiency at the gas–liquid interface. Experimental results (Movies S1 and S2) and Fig. 2A show that the microstreaming vortices around the acoustically actuated microbubble reach their peak intensity at the resonance frequency. Furthermore, the intensity of these microstreaming vortices increases with higher actuation voltages (Fig. 2B).

Simultaneously, simulations visualized the buoyancy-driven flow produced by the bubble’s ascent, which exhibits distinct macroscopic convection patterns as traced by microparticles. As depicted in Fig. 2D, when a strong acoustic stimulus is applied to a rising microbubble, the acoustically driven microstreaming markedly enhances local mass transfer around the bubble. Concurrently, the convection generated by the bubble’s buoyant rise expands the coverage area by over 3.5-fold compared to acoustic microstreaming alone, effectively coupling macroscale and microscale mass transfer (Fig. 2C and Movie S3). This synergistic effect of macro- and microscale mass transfer is key to the scalability of liquid manipulation: acoustic microstreaming intensifies mass transfer at the microscale under low Reynolds numbers, while the bubble’s ascent expands the macroscopic working space. Variations in the actuation voltage further influence the intensity and range of the flow field perturbation (Fig. 2D), providing an effective means to regulate the mixing process.

Simulation results also reveal that the flow field generated by an acoustically actuated rising microbubble (Fig. 2E) achieves a mean velocity amplitude 12 to 15 times greater than that of pure acoustic microstreaming, demonstrating a notable quantitative leap in mixing power, further demonstrating the superior mixing capability of this method (Movie S4). We also investigated the relationship between particle mixing intensity and acoustic actuation parameters (voltage and frequency), with results indicating that mass transfer efficiency can be flexibly controlled through the fine-tuning of these acoustic parameters (Fig. 2F and G). In summary, the proposed acoustically actuated rising microbubble method can generate large-scale flow fields while simultaneously enhancing microscale mass transfer, offering a highly efficient solution for various liquid manipulation scenarios. Applications include high-speed mixing of viscous fluids, acceleration of chemical synthesis, and enhancement of mass transfer efficiency in biomedical processes such as cell transfection and thrombolysis.

### Experimental setup

The orthogonal microscope setup included a handheld optical microscope (Hirox CX-10C, Hirox, China) with a lens and an industrial camera (MV-CU004-10GC, Hikrobotics, China) equipped with a 4- to 12-mm-focal-length manual zoom lens (MV-LB-4-12, MindVision, China), mounted on the side and bottom, respectively. The handheld optical microscope, used for side views, was connected to a high-speed movie camera (CP70-2-M-1000, Optronis, Germany) to record the bubble rising process at a frame rate of 5,500 FPS. A piezoelectric transducer, bonded to the space next to the container, emitted acoustic waves, with signals supplied by a function generator (AFG-2225, GW Instek, China). If necessary, the signal was amplified using a power amplifier (ATA-2022H, Aigtek, China). A 3-degree-of-freedom robotic arm (AUBO-E3, AUBO Robotics, China) was fixed next to the container to provide spatial positioning for the bubble-generating end. Microtubes, mounted on the actuating end of the robotic arm, were connected to a 10-ml syringe placed on a syringe pump (LSP02-2B, Longer, China), which controlled the size and speed of the generated bubbles.

### Microbubble and acoustic wave

The microbubbles used in all experiments were generated via a high-precision syringe pump system using a 10-μm inner-diameter glass capillary. The precise size distribution of the bubbles was characterized using high-speed microscopy and image analysis, revealing a mean diameter of $\bar{d}=120\;\mathrm{\mu m}$ with a size range of 90 to 150 μm. The acoustic field was generated by a piezoelectric transducer (PIC255, Physik Instrumente, China) operating at its specified resonance frequency (6.2 kHz). The transducer was permanently fixed to the bottom exterior of the experimental liquid container, centrally aligned, with its active surface positioned 1 mm from the base of the liquid column. The driving power for the acoustic actuation is reported as the input peak-to-peak voltage ($V_{\mathrm{pp}}$) applied to the transducer. This is a common practice in relative performance studies within customized microfluidic platforms, as the relationship between the applied voltage and the resulting acoustic pressure is highly dependent on the custom setup’s geometry and acoustic field complexity. All performance comparisons concerning acoustic intensity are strictly relative to the applied voltage within this defined experimental system.

Importantly, although increasing voltage may introduce mechanical vibrations at the macroscopic scale, such effects were minimized in our experimental design. The transducer and liquid container were mechanically secured using multiple layers of adhesive reinforcement (e.g., tape and structural stabilization of the holding frame) to suppress container-level oscillation and prevent vibration transmission to the bulk liquid. As a result, macroscopic mechanical vibration was negligible relative to the acoustic microstreaming generated at the bubble scale and was not observed to influence the flow patterns captured in the experiments. Therefore, the observed changes in mixing intensity with varying voltage originate from the modulation of acoustic driving strength on bubble oscillation, rather than from extraneous mechanical vibration. This is why the study focuses on voltage-driven acoustic effects, and mechanical vibration is not included as a separate parameter in the analysis.

## Results

### Efficient mixing in high-viscosity media

Conventional liquid mixing technologies are notoriously inefficient when processing high-viscosity fluids [4,23,55]. At low Reynolds numbers, large-scale mixers like bubble column reactors and magnetic stirrers struggle to achieve effective microscopic mass transfer. Conversely, while microfluidic chips can achieve microscale mixing, they often lack the throughput and volume required for industrial applications. Furthermore, traditional ultrasonic mixing typically relies on high-frequency shock waves and high power input [34,56], which is not ideal for many sensitive substances. Therefore, we propose a novel mixing method that combines tunable acoustic actuation with the rising motion of microbubbles, offering a low-energy and controllable alternative for the scalable and efficient mixing of high-viscosity fluids.

**Fig. 3.**
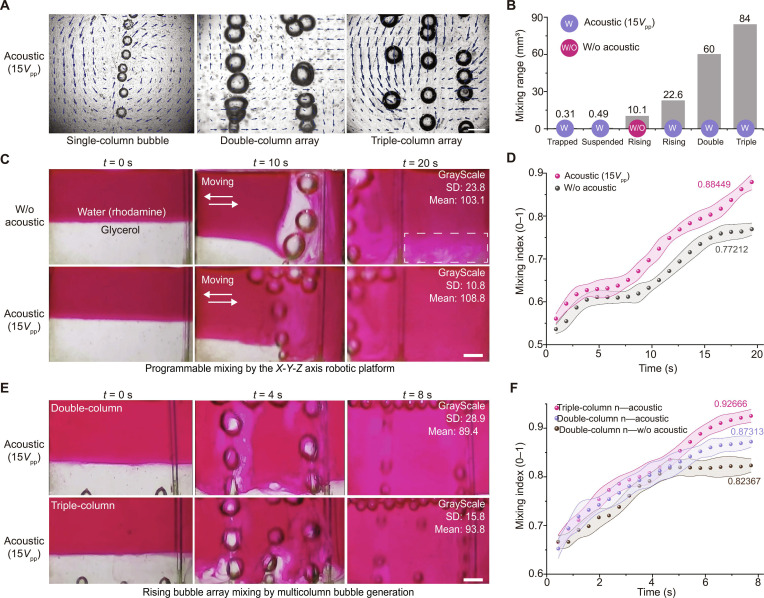
Acoustic rising bubbles enable efficient, high-throughput mixing of high-viscosity fluids. (A) Particle image velocimetry showing mixing by single-, double-, and triple-column bubble arrays with acoustic waves. Scale bar: 400 μm. (B) Comparison of mixing regions for different methods. (C) Pre-programmed motion of a single-column bubble mixing glycerol and rhodamine (red) solutions. Scale bar: 1 mm. (D) Mixing index for single-column moving bubble with and without acoustic waves. (E) Double- and triple-column bubble arrays mixing glycerol and rhodamine solutions. Scale bar: 1 mm. (F) Mixing index for various bubble arrays in high-viscosity fluids with and without acoustic waves. Shaded error bands represent the standard deviation (*n* = 3).

Experiments demonstrate that by superimposing buoyancy-driven convection with localized acoustic microstreaming, acoustically actuated microbubbles can disrupt laminar interfaces and enhance convective transport. Particle image velocimetry results show that compared to a single-column configuration, double- and triple-column microbubble arrays produce a significantly larger range of flow field perturbation and a broader mixing zone (Fig. 3A and B and Fig. S12). In a stratified solution of glycerol and rhodamine-dyed aqueous solution, a single column of acoustically actuated rising microbubbles achieved a mixing index of 88.4% ± 3.2% within 20 s, representing a 55% reduction (P<0.01) in mixing time compared to the passive control (Fig. 3C and D and Fig. S13). In contrast, a triple-column array achieved a mixing index of 92.7% ± 2.5% in just 8 s, resulting in a 100% enhancement (P<0.001) in efficiency compared to robot-assisted stirring (Fig. 3E and F, Fig. S14, and Movie S5). These results suggest that microbubble arrays have the potential to replace complex robotic mixing strategies for large-area liquid mixing (Fig. S15). To validate the practical utility of this method, we integrated it into a programmable platform that utilizes a robot to execute preset trajectories and evaluate mixing performance in real time (Figs. S16 to S18). Using this platform, we successfully mixed various fluid systems, such as oil-based and water-based inks (Fig. S19), alkaline solutions, and polystyrene particle suspensions with diameters ranging from 1 to 100 μm (Fig. S20). These cases validate the method’s feasibility for mixing fluids and particle suspensions with different properties, providing an experimental foundation for future scale-up in industrial and biomedical fields.

### Chemical process enhancement

In liquid-phase chemical synthesis, reaction rates are often substantially limited by slow mass transfer processes and restricted interfacial areas, especially in gas–liquid or high-viscosity systems [57,58]. Acoustic rising microbubbles overcome these limitations by combining buoyancy-driven convection with localized acoustic microstreaming effects. The acoustic microstreaming disrupts laminar boundary layers, expands the gas–liquid interfacial area, and generates vortices, thereby materially enhancing convective mass transfer and solute diffusion.

**Fig. 4.**
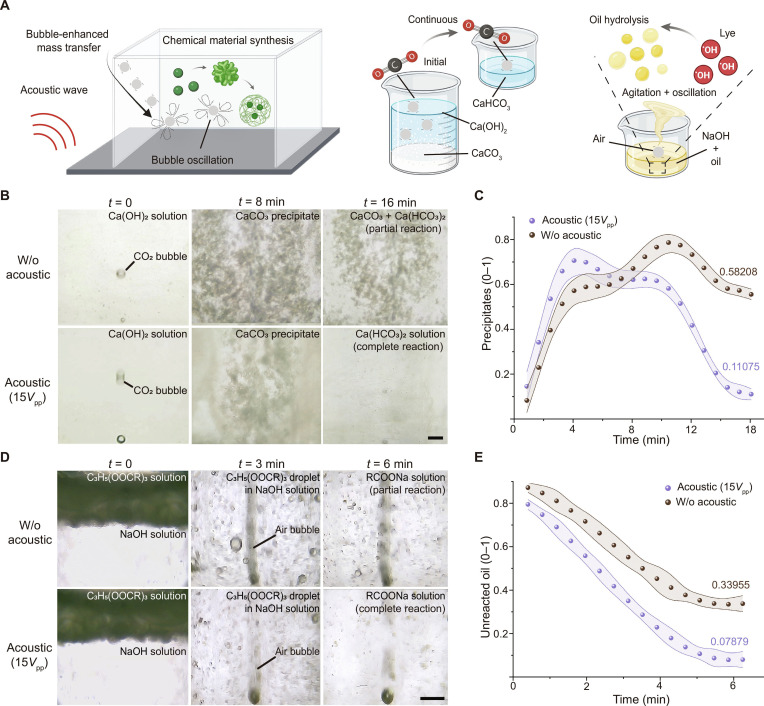
Acoustic rising bubbles accelerate chemical reactions. (A) Schematic of bubble-enhanced reactions including carbon dioxide in clarified limewater, and oil hydrolysis under alkaline conditions. (B) Production and dissolution of precipitates. Scale bar: 1 mm. (C) Precipitation over time with and without acoustic waves. (D) Saponification acceleration by rising bubble mixing. Scale bar: 1 mm. (E) Unreacted oil over time with and without acoustic waves. Shaded error bands represent the standard deviation (*n* = 3).

We validated this mass transfer enhancement mechanism in 2 representative reaction systems (Fig. 4A). In the precipitation reaction between carbon dioxide and calcium hydroxide, acoustically actuated rising microbubbles increased the degree of turbulence and gas solubility, leading to a higher concentration of carbon dioxide (CO_2_) in the liquid phase and thus accelerating the formation of calcium carbonate (CaCO_3_) (Fig. 4B and Movie S6). Compared to the control group, the acoustically actuated microbubbles notably increased the gas–liquid interfacial area, resulting in an approximately 45% increase in the precipitation reaction rate (Fig. 4C). In the saponification of triglycerides, although the microbubbles did not directly participate in the chemical reaction, the microstreaming vortices they produced markedly improved the mixing of the alkali solution and triglycerides. This disrupted the laminar interface and enhanced the diffusion rate of the reactants (Fig. 4D and Movie S6). Quantitative analysis showed a significant reduction in the amount of unreacted oil (Fig. 4E), further confirming that acoustically actuated rising microbubbles can substantially accelerate chemical synthesis processes. These results demonstrate that by enhancing mixing at the macroscale and intensifying mass transfer at the microscale, acoustically actuated rising microbubbles offer a low-energy mass transfer enhancement strategy suitable for various reaction systems, both with and without gas participation.

### Cell transfection and thrombolysis

Efficient gene delivery requires the transient poration of the cell membrane without causing cell damage [59,60]. Traditional transfection methods, such as electroporation or chemical reagents, often reduce cell viability or lack spatial targeting control [61]. In contrast, acoustic rising microbubbles offer a controllable and highly efficient transfection strategy. The acoustic microbubbles generate localized microstreaming and shear forces around cells, causing transient deformation of the cell membrane and the formation of pores, thereby allowing exogenous molecules to enter the cell interior [62].

**Fig. 5.**
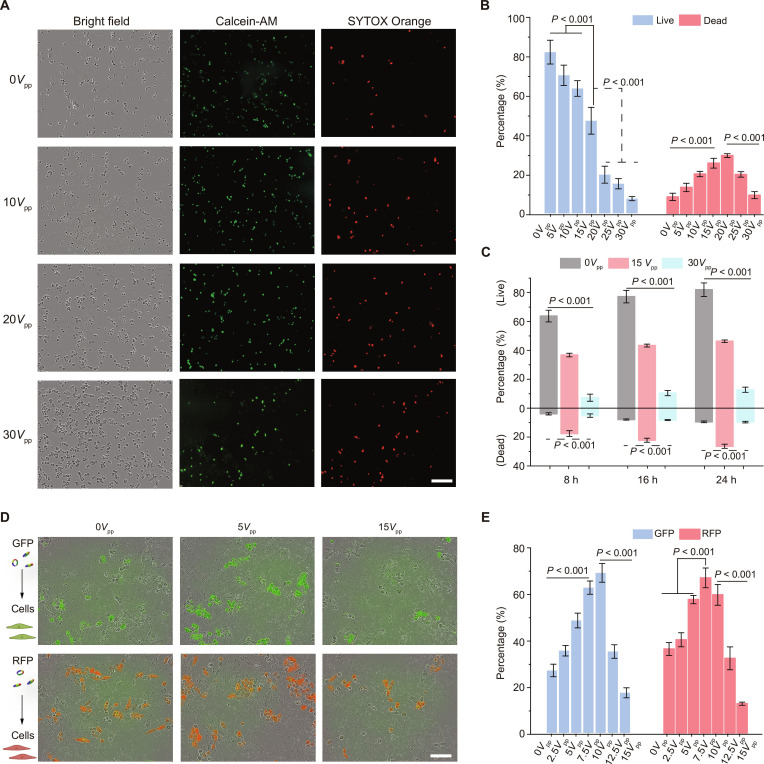
Acoustic rising bubbles affect cell viability and transfection. (A) Bright-field and fluorescence images of HeLa cells treated at various voltages. Scale bar: 400 μm. (B) Percentage of red (dead) and green (live) cells at each voltage. (C) Trends in live and dead cell percentages over 24 h after acoustic rising bubble treatment at different voltages. (D) Green/red fluorescence staining of transfected HeLa cells. Scale bar: 200 μm. (E) Percentage of green and red fluorescent cells among total cells in transfection. Error bars represent standard deviation (*n* = 3). GFP, green fluorescent protein; RFP, red fluorescent protein.

To evaluate the effectiveness of this method, we applied acoustic stimulation at different driving voltages to HeLa cells (Fig. 5A). Viability assays indicated that when the voltage exceeded 15*V*_pp_, the cell membrane suffered marked damage, leading to substantial cell death (Fig. 5B). However, at moderate voltages between 7.5*V*_pp_ and 10*V*_pp_, cell viability remained above 85%. Real-time imaging analysis (Fig. 5C) further revealed that the membrane pores induced by acoustic stimulation at moderate voltages were transient and did not cause long-term cytotoxicity. Under these conditions, the shear forces generated by the acoustically actuated microbubbles were sufficient to temporarily disrupt the cell membrane structure, allowing plasmids to enter the cells and achieve transfection (Fig. 5D). We observed that transfection efficiency reached a high level in the 7.5*V*_pp_ to 10*V*_pp_ voltage range, whereas at higher voltages, the efficiency declined due to extensive cell death (Fig. 5E). These results indicate that by precisely regulating the acoustic parameters, reversible cell membrane permeabilization can be achieved, thereby optimizing the transfection process. As long as the driving voltage is maintained below the cell lysis threshold, acoustic rising microbubbles can serve as a controllable gene delivery platform, achieving high transfection efficiency while ensuring high cell viability.

Thrombolysis and cell lysis both require localized mechanical forces to overcome structural barriers [63,64]. Conventional pharmaceutical or mechanical treatment methods are often slow or invasive. Acoustic rising microbubbles generate powerful microstreaming and shear stresses that create mechanical perturbations within a thrombus, altering the fibrin network structure and promoting its breakdown. Concurrently, these microbubbles can disrupt cell membranes, accelerating cell lysis.

**Fig. 6.**
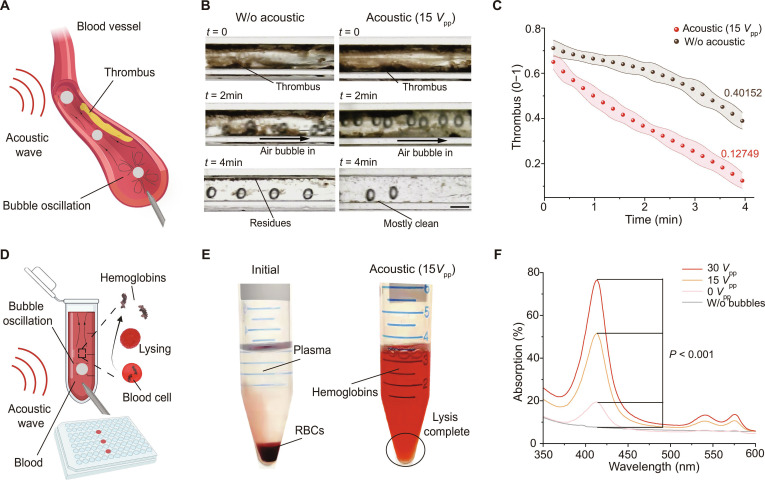
Acoustic rising bubbles enhance thrombus clearance and induce red blood cell (RBC) lysis. (A) Acoustic rising microbubbles in blood vessels for thrombus clearance. (B) Enhanced thrombus clearance with continuous bubbles and acoustic waves. Scale bar: 1 mm. (C) Thrombus clearance over time, showing improved thrombolysis with acoustic bubbles. Shaded error bands represent the standard deviation (*n* = 3). (D) RBC lysis by acoustic rising bubbles. (E) Hemoglobin is released into the blood after bubble treatment. (F) Ultraviolet–visible (UV–Vis) spectra of blood after rising bubble treatment at various driving voltages.

In our experiments, the microstreaming effect produced by acoustically actuated microbubbles effectively impinged on a fibrin network, causing mechanical fracture of the thrombus (Fig. 6A). Quantitative measurements showed a significant increase in the amount of thrombus cleared under acoustic stimulation (Fig. 6B and C and Movie S6), indicating a synergistic effect between mechanical perturbation and enhanced mass transfer. Furthermore, we observed a rapid lysis of mouse red blood cells when the driving voltage reached or exceeded 15*V*_pp_. The microstreaming and shock waves generated by the acoustically actuated microbubbles penetrated the red blood cell membranes (Fig. 6D), leading to the rapid release of hemoglobin into the supernatant. The supernatant of the treated group turned deep red and exhibited a distinct absorbance peak at 413 nm, whereas the hemoglobin content in the control group’s supernatant was negligible (Fig. 6E and F). The lysis efficiency increased with the driving voltage, demonstrating the tunable destructive capability of this method. These findings establish acoustically actuated rising microbubbles as a multifunctional platform, offering new possibilities for rapid and efficient biomedical applications such as thrombolysis and cell lysis through the synergistic action of mechanical disruption and acoustic microstreaming.

## Discussion

The efficient mixing of high-viscosity fluids has long been a challenge in industrial production and laboratory applications (Table 1). Conventional mixing devices, such as rotor–stator systems, impeller stirrers, and bubble column reactors, often require energy inputs exceeding 1 kW/m^3^ to agitate high-viscosity fluids [7,21,23]. This high energy consumption not only increases operational costs but can also lead to the thermal degradation of sensitive components in the reaction system, such as enzymes, polymers, and living cells [26,36]. In pharmaceutical manufacturing, prolonged mixing times for high-viscosity formulations can cause batch-to-batch variations and pose regulatory challenges [59,60,65]. Moreover, in high-viscosity environments, the motion of bubbles in a column is restricted and microscopic turbulence is attenuated [22,66], further reducing mixing uniformity. These factors underscore the urgent need for a new strategy that can achieve energy-efficient, precise, and scalable homogeneous mixing.

**Table 1.** Comparison of industrial and laboratory mixing methods. The differences between acoustic rising bubbles and classical mixing methods (corresponding production companies) including mechanical stirring, magnetic, ultrasonic, and bubble column reactors are compared in terms of operational range, capacity, application, and cost.

Method	Technique	Power consumption	Throughput	Mixing time (water) per unit volume (liter)
Magnetic stirrer (IKA MIXSTER)	Magnetic stir bar rotation under a magnetic field	30 W	600 μl	3–4 min
Shaker (Neuation)	Shaking action to mix and stir	400 W	250 × 5 μl	10–15 min
Ultrasonics (Anyland)	Cavitation generates microjets	80 W 2,400 W	50 μl 25 l	5–8 min
Bubble column reactor (KXmicroflow)	Buoyancy-driven flow and agitation	500 W	12 l	4–5 min
Mechanical stirring (EKATO)	Macroscopic flows (vortices)	7,500 W	500 l	20–25 min
Acoustic rising bubbles (this work)	Buoyancy-driven flow and mirostreaming	5 W	200 μl	20–40 s

Acoustically actuated rising microbubbles meet these needs by combining buoyancy-driven convection with acoustic microstreaming under resonance conditions. This approach enables the formation of intense microvortices at the microscale to disrupt laminar interfaces while markedly reducing energy consumption [67]. In the carbon dioxide–calcium hydroxide precipitation reaction, we observed a ~3.2-fold increase in the mass transfer coefficient, which shortened the required reaction time by 45% (Fig. 4C). In the saponification of triglycerides, our method achieved a 93.2% conversion rate within 4 min, whereas the control group required 6 min (Fig. 4E). These results demonstrate the method’s ability to simultaneously enhance chemical processes at both macroscopic and microscopic scales.

Beyond chemical synthesis, the tunable acoustic parameters of this method are also well suited for biomedical applications [63,64,68,69]. At a moderate voltage of 7.5*V*_pp_ to 10*V*_pp_, the bubble-induced shear forces can temporarily increase cell membrane permeability, achieving gene delivery with ~68% transfection efficiency while maintaining cell viability above 85% (Fig. 5D). At higher voltages (≥15*V*_pp_), the enhanced microstreaming and shock waves can disrupt the structure [62] of fibrin clots in blood (Fig. 6C) and substantially accelerate the lysis of mouse red blood cells (Fig. 6F), enabling rapid biomedical processing.

In conclusion, acoustically actuated rising microbubbles provide a sustainable, low-energy, and highly efficient method for liquid processing by combining macroscopic buoyancy-driven flow with localized acoustic microstreaming. This strategy applies to a wide range of operations in the chemical and biomedical fields. Through the fine-tuning of acoustic frequency and voltage parameters, this controllable microbubble system overcomes the high energy consumption of traditional industrial mixers and the limited throughput of microfluidic chips, offering a scalable, low-energy, and precise mass transfer solution [53]. The integration of this method with programmable robotic systems can further enable the automation and optimization of mixing processes. Future work will focus on optimizing acoustic parameter selection through computational fluid dynamics, developing scale-up laws for industrial reactors, and exploring more applications for this technology in fields such as advanced material synthesis and regenerative medicine.

## Data Availability

All data are available in the main text or the Supplementary Materials.

## Supplementary Materials

Supplementary Sections S1 to S4 Figs. S1 to S20 Movies S1 to S6 References [70–75]
