# Supplementary material for: Acoustic Rising Microbubbles for Efficient Liquid Operations
Source: Cyborg Bionic Syst. 2026 Mar 9;7:0449. doi: 10.34133/cbsystems.0449 (PMC12968397; doi:10.34133/cbsystems.0449)
Supplement: Supplementary file 1 — Supplementary Sections S1 to S4 Figs. S1 to S20 Movies S1 to S6 References [70–75] [file cbsystems.0449.f1.zip › SM revisions_cbsystems.0449 - 0204.docx]

Supplementary Information

**Acoustic rising microbubbles for efficient liquid operations**

Chenhao Bai^1^†, Zhuo Chen^1^†, Yunsheng Li^1^, Yan Chen^1^, Qing Shi^1^, Qiang Huang^1, 2^, Toshio Fukuda^3^, Tatsuo Arai^1, 4^, Xiaoming Liu^1, 5*^ ,

^1^School of Mechatronics Engineering, and Key Laboratory of Biomimetic Robots and Systems, Ministry of Education, Beijing Institute of Technology, Beijing, 100081, China.

^2^Thrust of Robotics and Autonomous Systems, The Hong Kong University of Science and Technology (Guangzhou), Guangzhou, 511453, China.

^3^Institute of Innovation for Future Society, Nagoya University, Nagoya, 4648601, Japan.

^4^Center for Neuroscience and Biomedical Engineering, The University of Electro-Communications, 1828585, Japan.

^5^School of Medical Engineering, Beijing Institute of Technology, Zhuhai, 519088, China.

†These authors contributed equally: Chenhao Bai, Zhuo Chen.

^*^Correspondence should be addressed to Xiaoming Liu ([liuxiaoming555@bit.edu.cn](mailto:liuxiaoming555@bit.edu.cn)).

This PDF file includes:

Supplementary Section S1-4

Supplementary Figure S1-20

Other Supplementary Materials for this manuscript:

Supplementary Movies 1-6

**Supplementary Section S1. Mixing effect based on agitation and oscillation**

The rising bubble process is characterized by the synergy of shear-induced turbulence (SIT), large-scale buoyancy-driven flow, and bubble-induced agitation (BIA) mixing, where rising bubbles create shear effects and interact with the fluid, enhancing mass transfer by increasing the surface area for gas-liquid interactions [74]. The rising bubble from the bottom of a container to the liquid surface induces significant pressure dynamics and vortices, which in turn generate turbulence and agitation that significantly improve mixing efficiency. Initially, it initiates a volume repulsion effect, characterized by the displacement of surrounding liquid to create agitation. Additionally, the shift in the velocity distribution of the surrounding fluid forms symmetric vortices near the rising region. Furthermore, it induces shear forces in the vicinity, leading to the formation of a pressure gradient, thereby promoting fluid mixing and diffusion. The mixing phenomena due to bubble rise comprises three fluid dynamics effects, which can be partially predicted by simple superposition：

$$\begin{aligned} \text{Mixing effect}\propto U_{b}\cdot U_{w}+\frac{E_{k}}{\rho}+\alpha\left( \frac{D_{m}}{d} \right)^{\frac{1}{2}} \# \left( 1 \right) \end{aligned}$$

BIA is highly effective in promoting mixing and the intense local turbulence created by bubbles enhances the mixing of different fluid components, leading to more uniform concentrations and improved reaction rates in chemical processes [75]. At the microscale, as bubbles rise through a liquid, they create wakes, regions of disturbed fluid flow, behind them. The rise velocity of a bubble in a liquid, influenced by factors such as bubble size, liquid viscosity, and buoyancy effects, can be used to estimate the velocity in the wake of a rising bubble based on the rise velocity and bubble diameter:

$$\begin{aligned} U_{b}\approx\sqrt{\frac{2gd}{C_{D}}}\# \left( 2 \right) \end{aligned}$$

$$\begin{aligned} U_{w}=U_{b}\left( \frac{d}{D} \right)^{n}\# \left( 3 \right) \end{aligned}$$

where $U_{b}$is the bubble rise velocity, $d$is the bubble diameter, $g$is the gravitational acceleration, $C_{D}$is the drag coefficient (which can depend on the Reynolds number and bubble shape), $D$is the characteristic length of the container, and$n$is an empirical constant, the rise velocity of a bubble in a liquid can be considered as a function of the bubble diameter and gravitational acceleration.

SIT occurs when there is a significant difference in velocity between adjacent layers of fluid. These velocity gradients create shear forces that can destabilize the flow and lead to turbulence. Common sources of velocity gradients include flow over surfaces, such as the walls of a chamber, and flow around objects, like bubbles or particles suspended in the fluid. The primary structures in SIT are eddies and vortices, which are swirling motions of fluid. These structures exhibit a wide range of sizes, from large, energy-containing eddies to smaller, dissipative scales. SIT enhances mixing by increasing the contact between different fluid layers, thereby facilitating more efficient diffusion of momentum, heat, and mass. The kinetic energy induced by SIT can be expressed as:

$$\begin{aligned} E_{k}=\frac{1}{2}\rho\left( u^{'2}+v^{'2}+w^{'2} \right) \# \left( 4 \right) \end{aligned}$$

where $u^{'}$, $v^{'}$, and $w^{'}$are the root mean square values of velocity fluctuations in the x-, y-, and z-directions, respectively. The increased surface area provided by rising bubbles, along with the enhanced agitation, significantly improves mass transfer rates. The mass transfer coefficient can be influenced by the surface area and the agitation caused by the bubbles:

$$\begin{aligned} k_{L}=\alpha\left( \frac{D_{m}}{d} \right)^{\frac{1}{2}} \# \left( 5 \right) \end{aligned}$$

where$\alpha$is a dimensionless constant, $D_{m}$is the molecular diffusivity, and$d$is the diameter of the bubble. This is particularly advantageous in processes such as chemical material synthesis with gas absorption, where the rapid transfer of reactant gas into the liquid phase is a critical step in accelerating the overall process.

**Supplementary Section S2. Buoyancy-driven flows in the whole fluid environment**

The primary driver of buoyancy-driven flows is the difference in density between various parts of the fluid. In bubbly flows, the density difference is created by the presence of gas bubbles in the liquid [76]. These bubbles, being less dense than the surrounding liquid, rise due to buoyancy, creating an upward flow. As bubbles rise, they create complex flow patterns within the fluid. These patterns include large-scale circulations and vortices, which contribute to mixing and agitation. The flow patterns are contingent upon the dimensions, distribution, and ascent velocity of the bubbles. The buoyancy-driven flows are influenced by the presence of boundaries, such as container walls or free surfaces. These boundaries can direct the flow, generate supplementary vortices, and augment the mixing. The interaction between rising bubbles and boundaries frequently results in secondary flows and intricate circulation patterns. The most conspicuous feature of buoyancy-driven flows is the robust vertical motion caused by the rising bubbles. This vertical motion is a crucial factor in the overall mixing and agitation of the fluid. The rising bubbles induce large-scale circulatory flows, whereby fluid moves upward in the center and downward along the sides. This circulation enhances the mixing of the fluid, promoting uniformity in temperature and concentration.

The rise velocity of a bubble in a fluid is influenced by various factors, particularly the fluid's viscosity. In high-viscosity materials, the bubble's rise rate is typically slower due to increased fluid resistance. Fluid density also plays a crucial role, as it affects the buoyancy of the bubble; lower fluid density results in greater buoyancy, thereby accelerating the bubble's ascent. In a controlled fluid environment, the rate of gas injection can also be manipulated to directly influence bubble formation rates and sizes. Higher gas flow rates generally produce larger bubbles, which ascend more rapidly due to their increased buoyancy.

The velocity at which a bubble rises significantly influences the level of disturbance in the surrounding fluid. This disturbance primarily stems from the wake region generated as the bubble ascends (Fig. S9). As the bubble moves upward, it displaces the surrounding fluid, creating turbulence and eddies. The intensity of this disturbance is closely linked to the bubble's rise velocity, size, and the physical properties of the fluid. A faster rise velocity results in a more intense disturbance, as the bubble carries more kinetic energy, which is transferred to the surrounding fluid, inducing stronger flows and turbulence. Typically, an increase in rise velocity causes a transition in the fluid flow state from laminar to turbulent, a shift that can be characterized by the Reynolds number (Re). The Reynolds number, which depends on the rise velocity, bubble size, and fluid viscosity, predicts the likelihood of stronger turbulence and perturbations, which are essential for enhanced fluid mixing and mass transfer efficiency.

In practical applications such as chemical reactors, gas-liquid reaction units, or cell process systems in biotechnology, the bubble rise rate and the resulting perturbations directly affect substance mixing, mass transfer efficiency, and overall reaction rates. Therefore, by precisely controlling the bubble rise rate, the hydrodynamic behavior of these processes can be optimized, significantly improving working efficiency (Fig. S10). Moreover, strong perturbations increase the frequency of interfacial renewal between the bubble surface and the surrounding liquid, thereby enhancing the mass transfer rate between the gas and liquid phases. This effect is particularly important in high-viscosity materials, where enhanced perturbation not only overcomes viscous resistance but also promotes the uniform distribution of reactants, reduces concentration gradients, and ultimately accelerates the chemical material synthesis rate. Hence, understanding and effectively controlling the bubble rise rate is crucial for optimizing system performance in both industrial and scientific applications.

**Supplementary Section S3. Acoustic bubble oscillation principle**

The acoustic bubbles drive microstreaming, and when the frequency of the excited acoustic field matches that of the bubble, the maximum bubble oscillation amplitude is achieved [77]. The resonance frequency of a bubble in a liquid, also known as the Minnaert frequency, is indeed a key concept in the study of acoustic bubbles [78]. The formula is given by:

$$\begin{aligned} f_{0}=\frac{1}{2\pi R_{0}}\sqrt{\frac{3\gamma P_{0}}{\rho}}\# \left( 6 \right) \end{aligned}$$

where$f_{0}$is the resonance frequency, $R_{0}$ is the equilibrium radius of the bubble (the size of the bubble when it is not oscillating for adiabatic processes), $\gamma$ is the polytropic index meaning the ratio of specific heats, which depends on the process ($\gamma$ is typically 1.4 for air), $P_{0}$ is the ambient pressure, $\rho$ is the liquid density. By matching the frequency of the acoustic wave with that of the bubble's natural frequency, the generation of microstreaming can be enhanced. The simulation illustrates the high-speed flow around the bubble and the diverse effects of induced acoustic flow, which are crucial for achieving rapid and adequate micromixing.

The vortices generated by the acoustic secondary radiation force alter the surroundings, which are similar in size to the bubbles, inducing high-speed streamlines. A single bubble acts as a source to emit acoustic waves in all directions. The range of effect and velocity distribution of bubble-driven microstreaming vary with acoustic frequency, and selecting the optimal oscillation frequency can significantly enhance mixing efficiency. The simulation demonstrates that rising acoustic bubbles also engender a high-velocity flow field in their vicinity, enabling extensive mixing throughout the fluid environment due to their spatial advantages (Fig. S11). Driven periodically by acoustic waves, microbubbles respond to pressure and typically undergo volumetric oscillations or mutual vibration along an axis. When bubbles confined in a liquid encounter high-frequency acoustic waves, they induce bubble-driven microstreaming. These bubbles experience cycles of compression and rarefaction due to the alternating pressure of acoustic waves, which results in either contraction or expansion of the bubble size. This periodic expansion and contraction generate microstreaming around the bubble, which is contingent upon the frequency and amplitude of the acoustic wave, the bubble size, and the liquid properties (density and viscosity). The Rayleigh-Plesetter-Noordink-Nipiras-Polisky equation is often used to quantify these factors:

$$\begin{aligned} R\ddot{R}+\frac{3}{2}\dot{R}^{2}=\frac{1}{\rho}\left[ \left( P_{0}+\frac{2\sigma}{R_{0}}-P_{\nu} \right)\left( \frac{R_{0}}{R} \right)^{3\kappa}-\frac{2\sigma}{R}-\frac{4\mu\dot{R}}{R}-P_{0}-P\left( t \right) \right] \#\left( 7 \right) \end{aligned}$$

where $R_{0}$ is the initial bubble radius, $P_{0}$ is the hydrostatic pressure of the liquid, $P_{\nu}$ is the vapor pressure, $\rho$ is the liquid density, $\mu$ is the viscosity, $\sigma$ is the surface tension, and $\kappa$ is the polytropic index of the gas within the bubble.$P\left( t \right)$is time-varying ultrasound pressure. The distribution of the standing-wave field is described as

$$\begin{aligned} P\left( z,t \right)=P_{0}-2P_{A}\cos\left( \mathrm{kz} \right)\cos\left( \omega t+\phi_{0} \right)\#\left( 8 \right) \end{aligned}$$

where $P_{A}$ is the amplitude, $\omega$ is the frequency, $k$ is the wave number, $z$ is the space axis, and $\phi_{0}$ is the initial phase. When a bubble in the liquid is subjected to a periodic sound field, a type of acoustic radiation force caused by an external sound field acts on Bjerknes force.

The continuous acoustic rising bubble method involves the continuous generation and introduction of bubbles into a fluid, creating a sequence of oscillating bubbles under acoustic wave stimulation. These bubbles induce agitation and shear in the surrounding fluid, thereby enhancing mixing and mass transfer. Here, the rising bubbles effectively mix the entire fluid vertically within the container, while each bubble efficiently scatters sound, amplifying acoustic pressure in the fluid. This principle significantly accelerates mixing in high-viscosity materials and high mixing throughout applications, ensuring comprehensive mixing. Furthermore, its seamless integration into existing devices allows for efficient automated mixing, particularly in robot-assisted micro-mixing applications, enabling rapid and thorough mixing across scales from macroscopic to microscopic levels.

**Supplementary Section S4. Materials and experimental procedures**

Glass micropipette: BJ-40 capillary glass tubes were purchased from Jitian Bio, Beijing, China.

Polystyrene microspheres and microbeads: 1$\mu m$ and 10 $\mu m$ diameter microspheres purchased from BaseLine, Tianjin, China. 50$\mu m$ and 100 $\mu m$ diameter microbeads purchased from Duke Scientific, USA.

Chemical and biomedical reagents: Calcium hydroxide, sodium hydroxide, and anhydrous ethanol are all purchased from Aladdin, Shanghai, China. 99.9% pure carbon dioxide purchased from Yigas, Guangzhou, China. The ester used in the saponification reaction is commonly consumed as vegetable oil. tPA was purchased from Merck (USA). Xfect Transfection Reagent 631318 was purchased from Takara, Kyoto, Japan. Calcein-AM/PI staining kit was purchased from Solarbio, Beijing, China.

Cell sources: The HeLa cervical cancer cells were purchased from American Type Culture Collection (ATCC, USA). Mouse blood cells were kindly donated by Dr. Dong from Chinese Academy of Medical Sciences, Institute of Hematology (Tianjin). Deoxyribonucleic acid (DNA) plasmid was kindly donated by Dr. Liu from Beijing Institute of Technology (BIT, China).

HeLa cells: HeLa cells were cultivated in Dulbecco's modified Eagle's medium (DMEM). All the cell culture media were supplemented with 10% fetal bovine serum (Gibco, USA), 100 U/mL penicillin, 100μg/mL streptomycin, and 1x GlutaMAX (Gibco, USA). All cells were maintained in a humidified incubator at 37 ℃ with 5% CO2.

Thrombus formation: The thrombosis was formed via the previous protocol (73). Dispense 100 µL of fresh mouse blood into several plastic microtubes containing 50 U thrombin solution. These plastic microtubes are left at 37 ℃ for 3 hours and then moved to 4 ℃ for 3 days.

Polystyrene particles: The suspension needs to be diluted to 0.2% w/v with pure water, with shaking for mixing well before use. In the experiments of particle dispersion, polystyrene particles of 1 and 10 $\mu m$ diameter without any treatment were placed into 10 mL of pure water to form a 1% w/v suspension, which was used after full immersion. Polystyrene microbeads with 50 and 100$\mu m$ in diameter and 1.05 g/cm3 in density were diluted with pure water to the desired concentration. Using the proposed method, they were used in streamlined representation experiments for the single bubble and multiple column bubbles rising process.

Evaluation of cell viability: Wall-adherent HeLa cells were first digested using 0.25% trypsin-EDTA and collected. The cells were then washed with 1x PBS buffer, centrifuged at 450 g for 5 minutes, and washed twice with PBS buffer to remove trypsin and residual esterase. Next, the cell precipitates were resuspended and inoculated into the corresponding wells of 96-well plates, ensuring a concentration of 10⁵ to 10⁶ cells/ml in each well. They were cultured under suitable conditions, and cell growth was monitored. Once cell confluence reached 80%, fresh PBS buffer was added, and then acoustic rising microbubbles worked in the well, with different wells experiencing continuous bubble rising for 10 seconds at 5-second intervals, driven by acoustic waves of varying frequencies and voltages. Subsequently, 1-2 $\mu$L of calcein-AM and 3-5 µL of PI were added per mL of cell suspension, which was then placed in an incubator for long-term observation using the observing systems (Incucyte S2 Live-Cell Analysis System, Sartorius, Germany). The result was recorded at 1-hour intervals for 24 hours. Calcein-AM does not fluoresce until converted to calcein by intracellular esterases, emitting green fluorescence (Ex = 490 nm, Em = 515 nm) in living cells with active esterases. In contrast, PI (propidium iodide) does not penetrate the intact cell membrane of living cells and binds to the DNA of dead cells, emitting red fluorescence (Ex = 535 nm, Em = 617 nm).

Evaluation of cell transfection: 5 µg of plasmid DNA was first diluted with Xfect reaction buffer to a final volume of 100 µl in a microcentrifuge tube. The Xfect polymer was then added at a ratio of 0.3 µl per 1 µg of plasmid DNA and mixed by vortexing at high speed for 5 seconds. The mixture was incubated at room temperature for 10 minutes to form nanoparticle complexes for transfection. One day before transfection, wall-adherent HeLa cells were seeded into a 96-well plate with an appropriate amount of fresh cell culture medium. The cells were incubated in suitable conditions and monitored for growth. When cell confluence reached 50-70%, fresh phosphate-buffered saline (PBS) was added. The cells were then subjected to continuous bubble rising for 10 seconds at 5-second intervals, driven by acoustic waves of varying frequencies and voltages. After this treatment, the nanoparticle complexes were added to the cells, which were then returned to the incubator to allow gene expression. Transfections were recorded at 1-hour intervals for 24 hours using the observing systems (Incucyte S2 Live-Cell Analysis System, Sartorius, Germany). Finally, the cells were removed and observed using a confocal microscope (Nikon C2, Japan).

Evaluation of cell lysis: Mouse blood cells were obtained from the Institute of Hematology, Chinese Academy of Medical Sciences (Tianjin, China) and diluted tenfold with phosphate-buffered saline (PBS) at room temperature. The samples were then transferred to centrifuge tubes and subjected to continuous bubble rising for 10 seconds at 5-second intervals using a bubble generator device, driven by acoustic waves of varying frequencies and voltages. After the red blood cells were lysed, their internal hemoglobin was released into the solution, resulting in a distinct deep red color in the supernatant of the treated samples, while the supernatant of the untreated samples showed almost no signs of hemoglobin. To further quantify the released hemoglobin, the lysed samples were centrifuged at 800 rpm for 10 minutes to remove any remaining unlysed cells, a critical step for eliminating target proteins and nucleic acids from unlysed cells. The supernatant was then transferred to a 96-well plate for hemoglobin analysis using a full-wavelength spectrophotometer (FlexA-200, Allsheng, China). The absorbance of all samples was measured under different experimental conditions, and a significant increase in peak intensity at 413 nm confirmed the lysis of erythrocytes.

Evaluation of thrombus clearance: The microtubes containing thrombi were removed from a 37 ℃ incubator and quickly transferred to the experimental platform for thrombus dissolution. A piezoelectric transducer was attached to one side of the microtubes, generating acoustic waves inside the tubes using a sinusoidal voltage of 6.2 kHz and 15 Vpp. One end of the microtubes was submerged in fresh PBS buffer containing 0.1 mg/kg of tPA, while the other end was connected to a hose. A peristaltic pump was used to slowly deliver PBS buffer with 0.1 mg/kg of tPA into the tubes from a reservoir, facilitating thrombus removal. Bubbles were generated at the submerged orifice for debulking. The outflow was collected in a waste pool connected to the other end of the hose. A second microtube was subjected to the same experimental setup, but fresh PBS buffer without tPA was used while applying the same voltage. The control group underwent the experiment under identical conditions but without the application of voltage. Thrombus dissolution in the microtubes was recorded over 5 minutes using side-view microscopy, and the extent of thrombus removal was quantified using image processing techniques.

Quantify mixing efficiency: To quantitatively examine and evaluate the mixing efficiency, the mixing index was calculated using the following equation:

$$\begin{aligned} \left\{ \begin{aligned} M_{k}=1-\frac{V_{k}}{V_{0}} \\ V_{k}=\frac{\sqrt{\frac{1}{n}\sum\left( C_{i}-C_{m} \right)^{2}}}{C_{m}} \end{aligned} \right.\#(9) \end{aligned}$$

where $M_{k}$ is the mixing efficiency at moment $k$, $V$ is the coefficient of variation, and $V_{0}$ is the initial moment, $C_{i}$ is the grayscale value of each pixel, $C_{m}$ is the average value, and $n$ is the total number of pixels. With this definition, the calculation results in 0 for no mixing and 1 for complete mixing.

Statistical analysis: All quantitative experiments, including mixing performance, reaction rate enhancement, and cell viability assays, were performed independently for a minimum of $N$ $=3$ times. The results are presented as the mean value $\pm$ the standard deviation ($\mathrm{SD}$). This inclusion of error bars in all quantitative figures visually represents the data variability and reliability of the reported performance enhancements. Statistical comparisons between different groups were performed using One-way Analysis of Variance ($\text{ANOVA}$) to test the hypothesis of significant differences in means. When the ANOVA test yielded a significant result ($\text{p<0.05}$), subsequent pairwise comparisons were conducted using Tukey’s Post-hoc test to identify which specific groups showed statistically significant performance differences. A difference was considered statistically significant at a $\text{p-value<0.05}$ (or $\text{p-value<0.01}$ where noted).


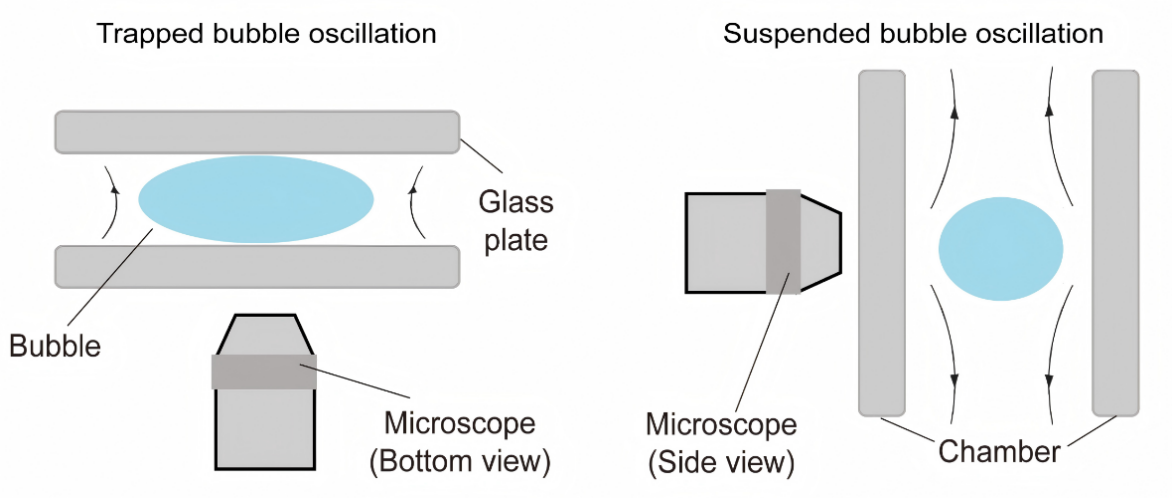


**Fig. S1. Analysis of the flow field generated by acoustic bubble oscillation under different observation conditions. Schematic illustrating the observation method and positioning for trapped (2D planar) and suspended (3D stereoscopic) acoustic bubbles. Trapped bubbles were confined between two 1 mm thick glass slides, while suspended bubbles were observed in a 10 mm thick chamber. This setup allows for the characterization of bubble-driven microstreaming and its mixing region under varying constraint levels.**


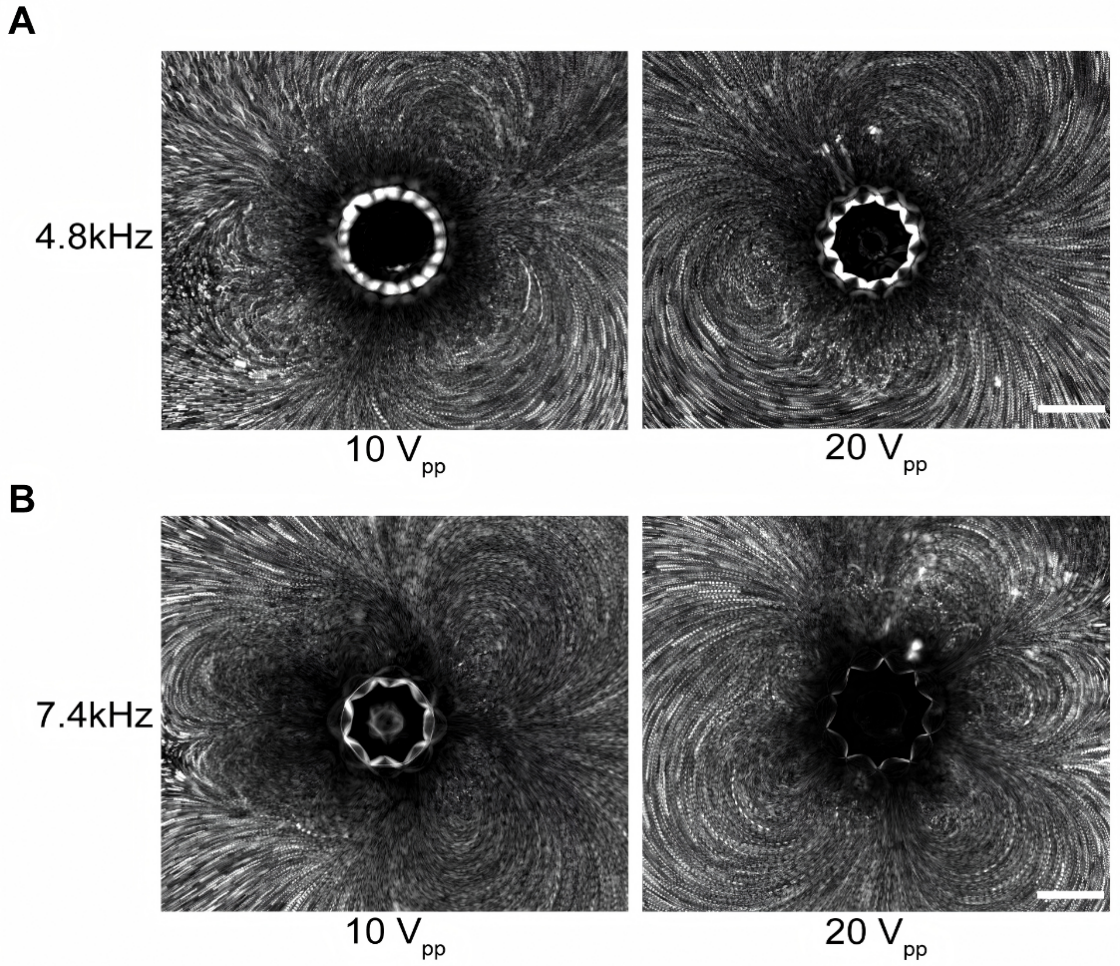


**Fig. S2. Streamlines of trapped bubble oscillation at non-resonance frequencies. Corresponding streamline distributions from Particle Image Velocimetry (PIV) analysis of trapped acoustic bubbles at 4.8 kHz and 7.4 kHz (non-resonance frequencies), showing the oscillation results at different driving voltages. Compared to the resonance frequency flow field, these exhibit weaker flow intensity and less effective mixing. Scale bar: 200** $\mu m$**.**


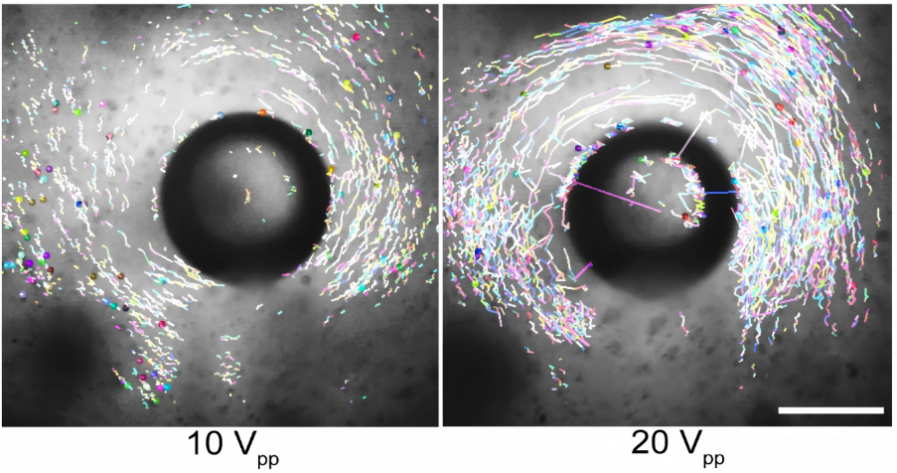


**Fig. S3. Figure S3. Mixing effect of suspended bubble oscillation under different voltages at the resonance frequency (6.2 kHz). The movement paths of particles affected by bubble oscillations were traced using an optical flow algorithm. This figure demonstrates that the intensity of the flow field generated by suspended bubble oscillation increases significantly with the driving voltage. Higher voltages force particles near the bubble to move faster and more effectively, achieving stronger localized mixing. Scale bar: 200** $\mu m$**.**


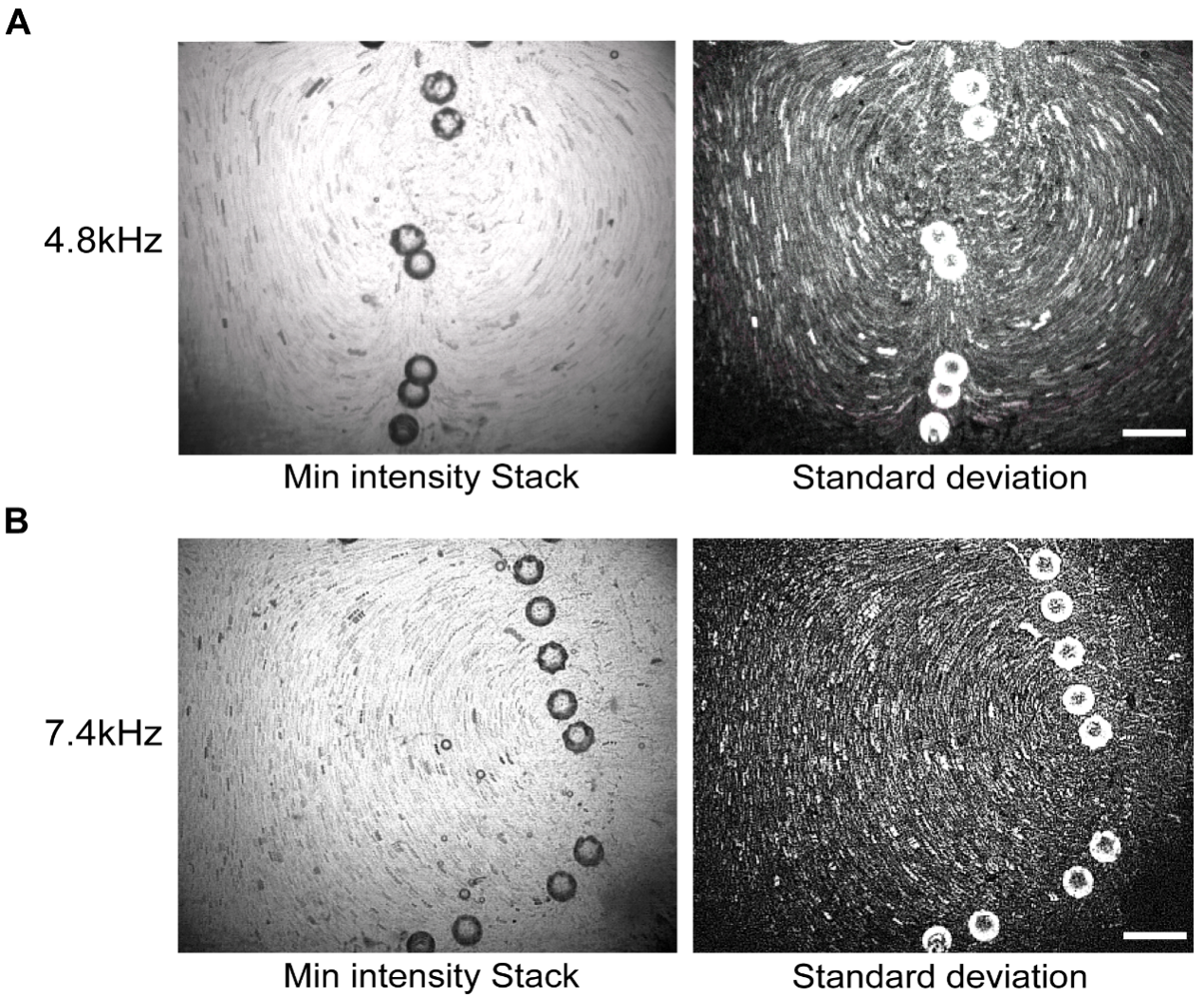


**Fig. S4. Streamlines of acoustic rising bubbles at non-resonance frequencies. The Min-intensity stack, obtained by stacking 10 images of the acoustic bubble rising process captured at 6000 FPS using the minimum value method in ImageJ, and the Standard Variance stacking method clearly characterize the mixing effect around the acoustic rising bubbles at non-resonance frequencies. The results indicate that both the flow field intensity and the mixing region are substantially reduced compared to the resonance frequency. Scale bar: 1 mm.**


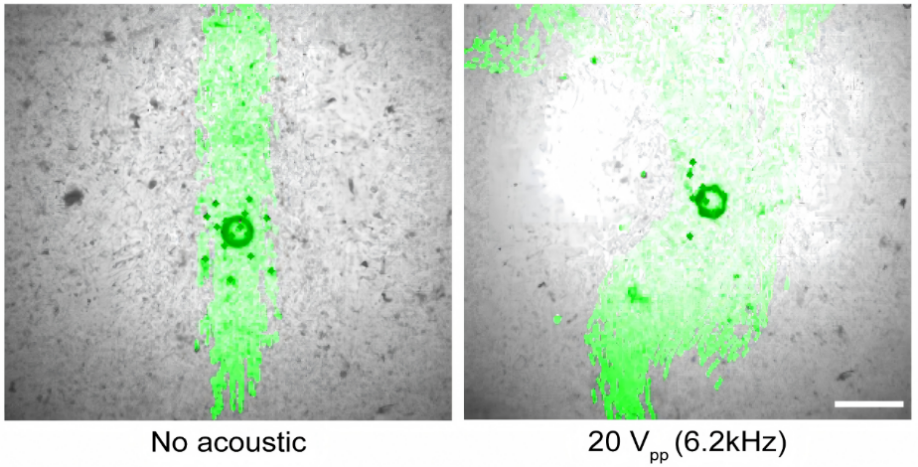


**Fig. S5. Acoustic rising bubbles can extend the mixing region compared to a normal rising bubble without acoustic waves. By utilizing a high-speed camera (6000 FPS) to record the rising process and tracing particle movement around the bubble via optical flow algorithms, the acoustic rising bubble is shown to have a significantly larger region of influence. This highlights its ability to enhance mixing across macroscopic scales. Scale bar: 1 mm.**


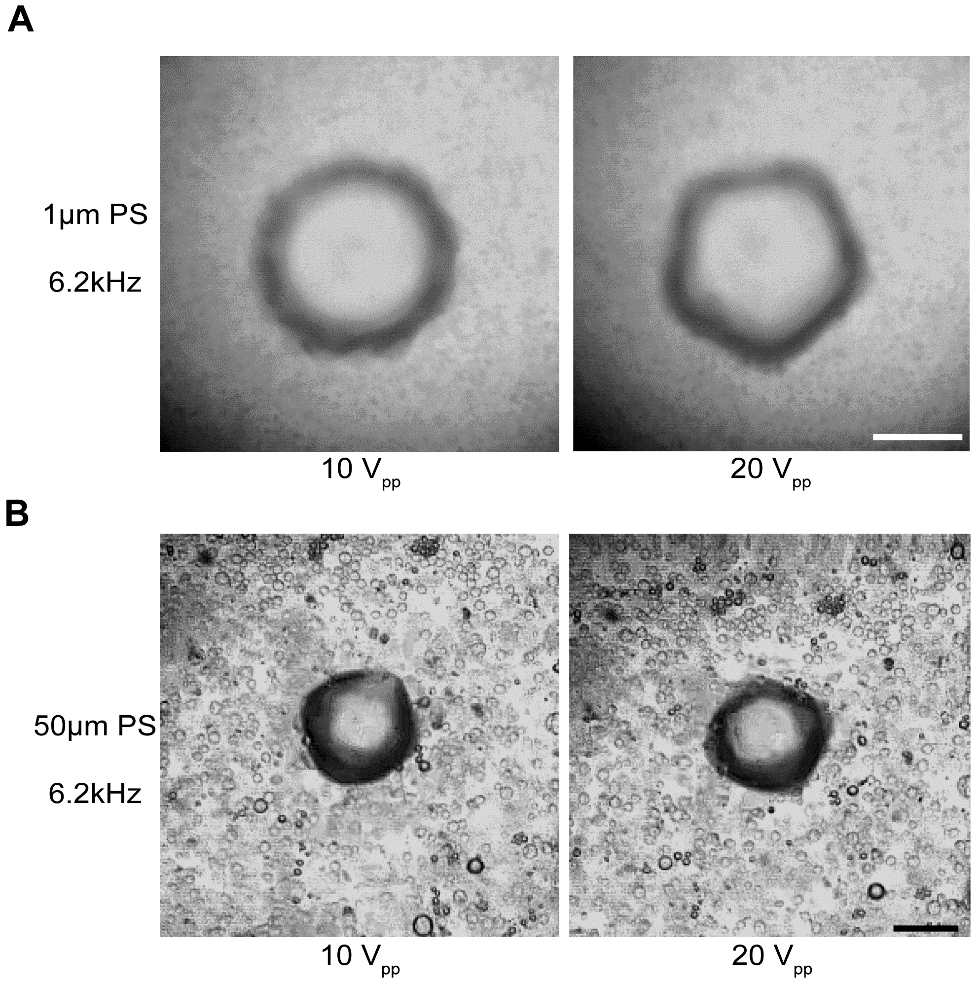


**Fig. S6. Dispersing effect of acoustic bubbles on Polystyrene (PS) particles of different diameter sizes at the resonance frequency (6.2 kHz). Experimental images confirm that acoustic bubbles at the resonance frequency (6.2 kHz) demonstrate effective localized dispersion for both 1** $\mu m$ **and 50** $\mu m$ **diameter PS particles in water. The dispersing effect is observed to strengthen as the driving voltage increases. Scale bar: 200** $\mu m$**.**


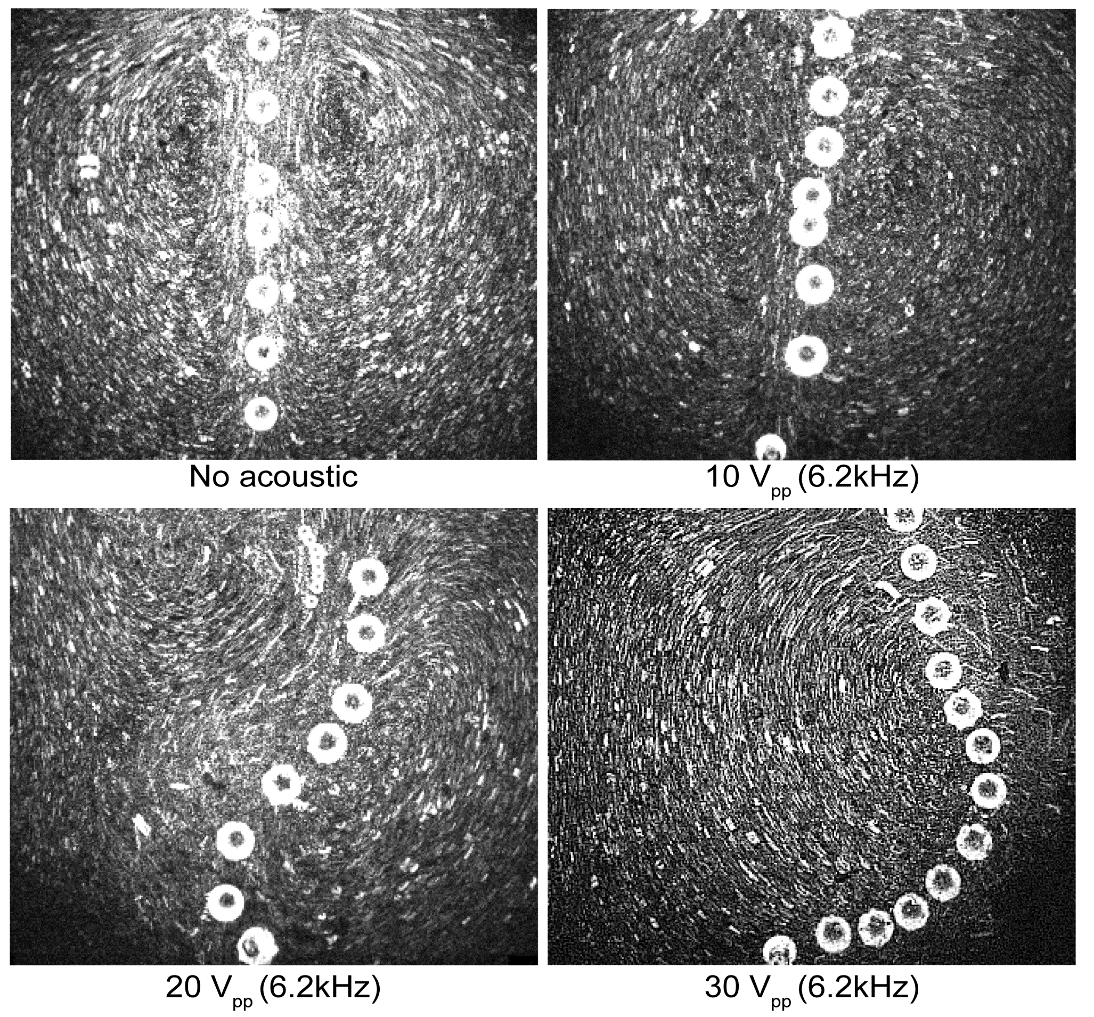


**Fig. S7. Standard variance stack of the rising bubble in a 1** $\boldsymbol{\mu m}$ **diameter PS particle environment driven by various voltages at the resonance frequency (6.2 kHz). Using the Standard Variance stacking method, this figure quantitatively shows that acoustic rising bubbles generate robust fluid interactions and exhibit a significant macroscopic dispersion effect compared to normal rising bubbles without acoustic waves. The results confirm that increasing the voltage enhances this dispersion effect. Scale bar: 1 mm.**


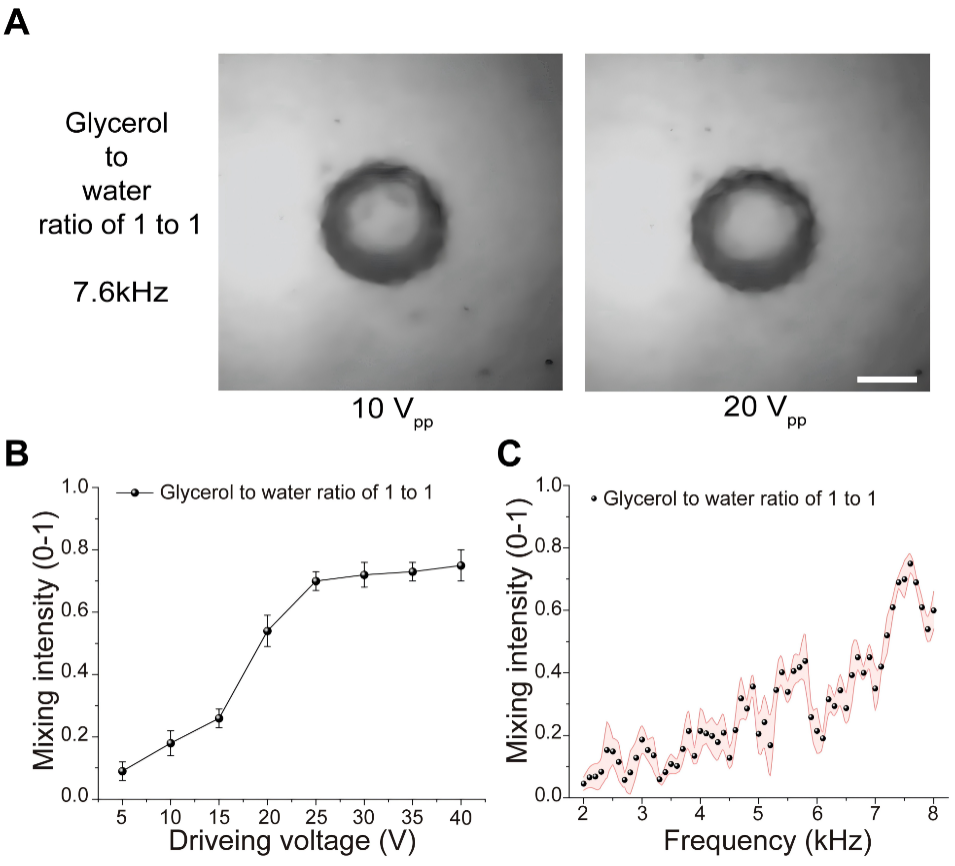


**Fig. S8. Acoustic bubble oscillation in high-viscosity materials (glycerol-to-water ratio of 1:1). Experimental results show that fluid viscosity influences the bubble's resonance frequency, with the maximum response frequency determined to be 7.6 kHz in this high-viscosity fluid. The enhancement of the mixing effect by increasing the voltage was also investigated, demonstrating that the enhancement is most significant at 25 V_pp_. Scale bar: 200**$\text{ μm}$**.**


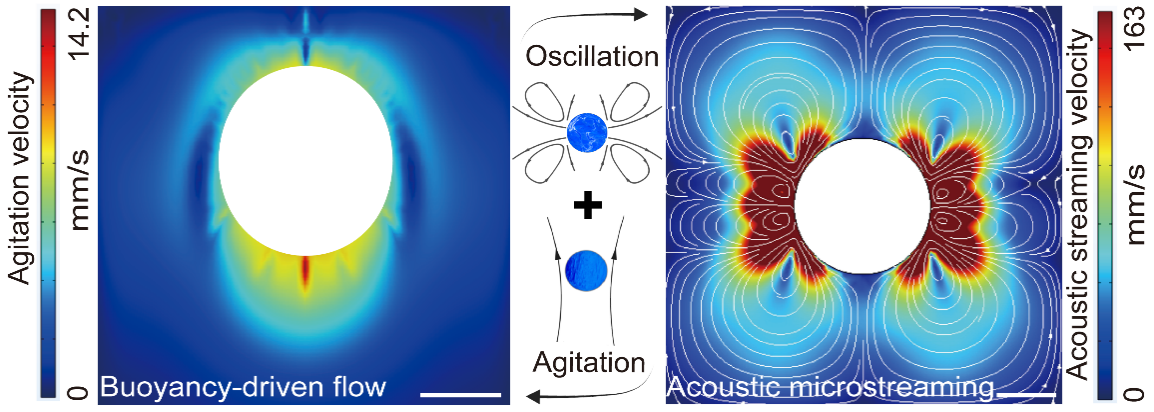


**Fig. S9. Effects of buoyancy-driven flow induced by rising bubbles and acoustic bubble oscillation microstreaming at the driving voltage of 30 V_pp_. Computational Fluid Dynamics (CFD) simulation separately illustrates the large-scale buoyancy-driven convection induced by a plain rising bubble and the localized microstreaming generated by an acoustic bubble oscillation. Investigating these two flow types individually helps to understand how their superposition in the acoustic rising microbubble achieves efficient mass transfer. Scale bar: 200**$\text{ μm}$**.**


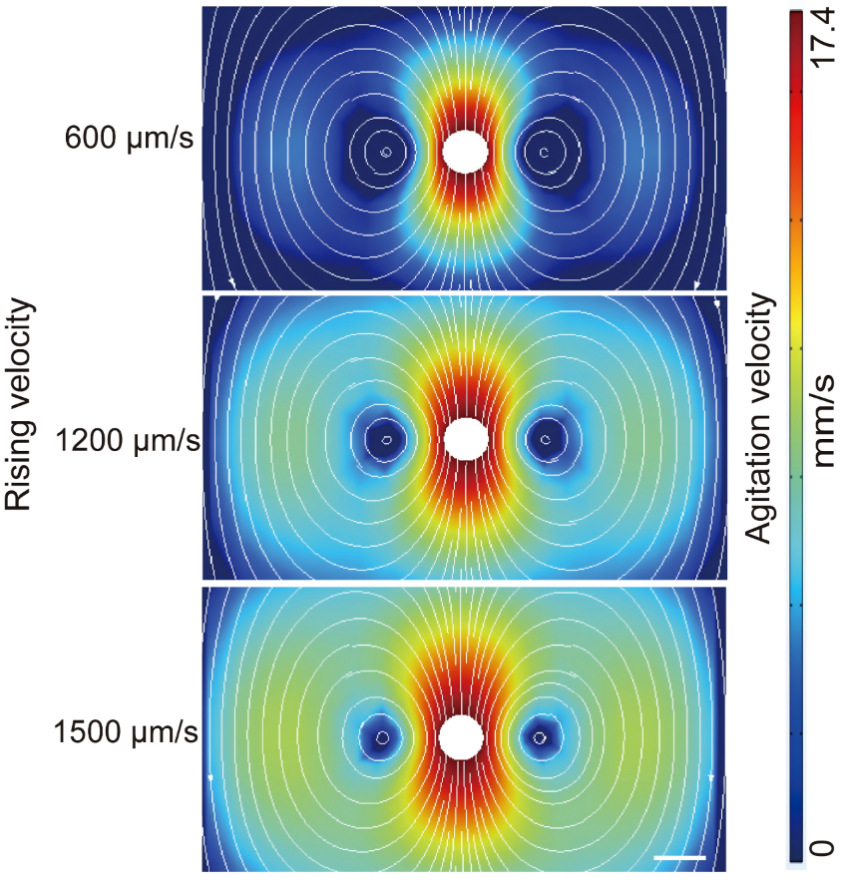


**Fig. S10. The effect of rising velocities on buoyancy-driven flows of rising bubbles. Simulation results show that the bubble's rising velocity significantly impacts the surrounding flow field. Faster rise speeds lead to a larger mixing region and more intense agitation, transferring greater kinetic energy to the surrounding fluid and inducing stronger flows and turbulence. Scale bar: 500**$\text{μm}$**.**


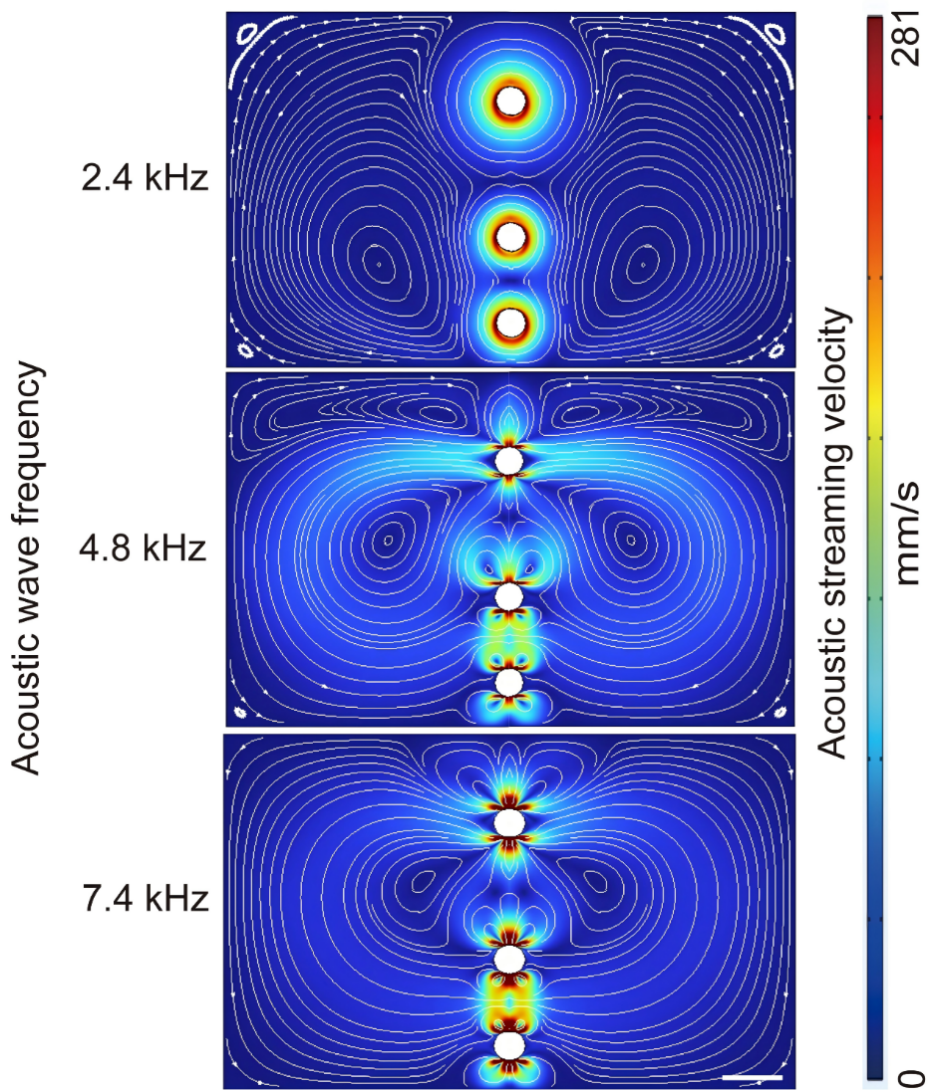


**Fig. S11.** **The effect of frequencies on the localized microstreaming surrounding the acoustic rising bubbles. Simulation results indicate that while the velocity of streaming generated around acoustic rising bubbles generally increases with frequency, the maximum mixing region is only achieved at the resonance frequency (6.2 kHz). This highlights the criticality of selecting the optimal oscillation frequency for maximum mixing efficiency. Scale bar: 1**$\mathrm{mm}$**.**


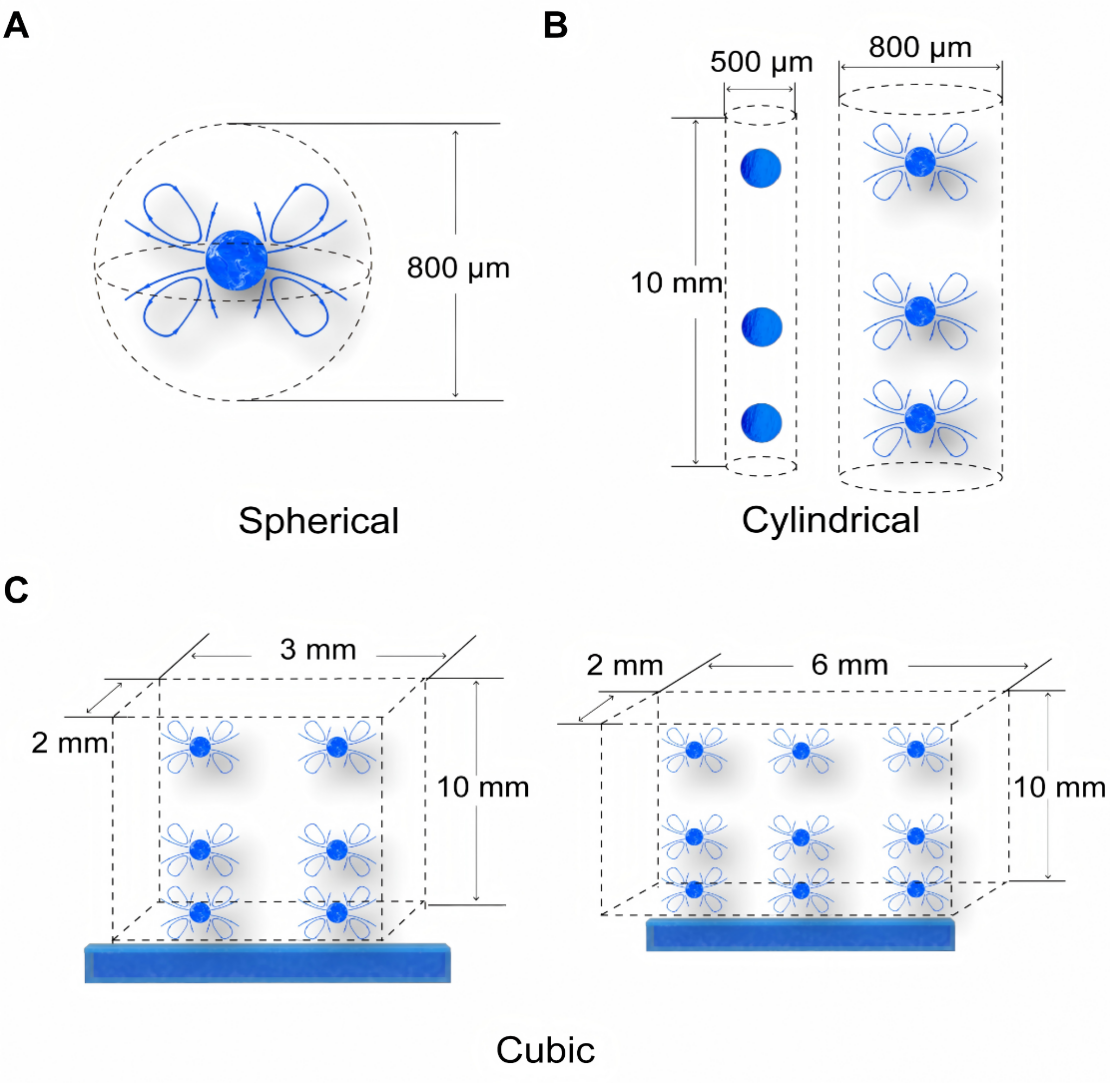


**Fig. S12. The mixing region of suspended acoustic bubble oscillations, normal rising bubbles, acoustic rising bubbles, and acoustic rising bubble arrays. Simulations and experiments reveal that suspended bubble oscillations generate a roughly spherical range, whereas the region of normal rising bubbles is cylindrical. In contrast, acoustic rising bubbles produce a cylindrical region with an enlarged bottom area. Furthermore, the mixing region of an acoustic rising bubble array approximates a cubic shape, which can be expanded further by increasing the number of bubble columns.**


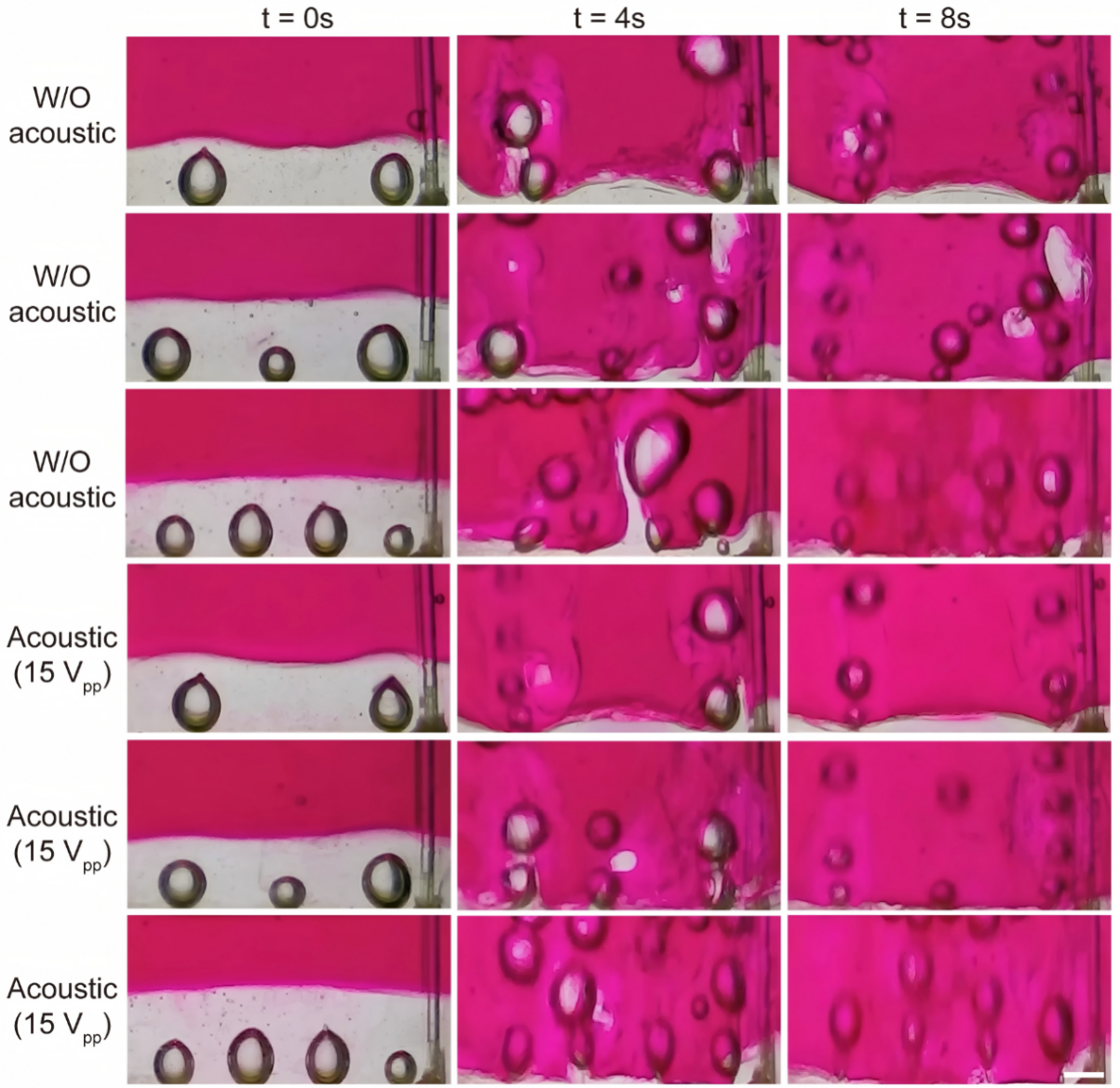


**Fig. S13. Acoustic rising bubble arrays (double-column, triple-column, and quadruple-column) in mixing high-viscosity materials (glycerol-to-water ratio of 1:1). This figure demonstrates that increasing the number of bubble columns allows the acoustic rising bubble array to achieve more homogeneous and complete mixing. Image comparison also shows that even in the absence of an acoustic field, increasing the number of columns significantly affects the mixing outcome. Scale bar: 1**$\mathrm{mm}$**.**


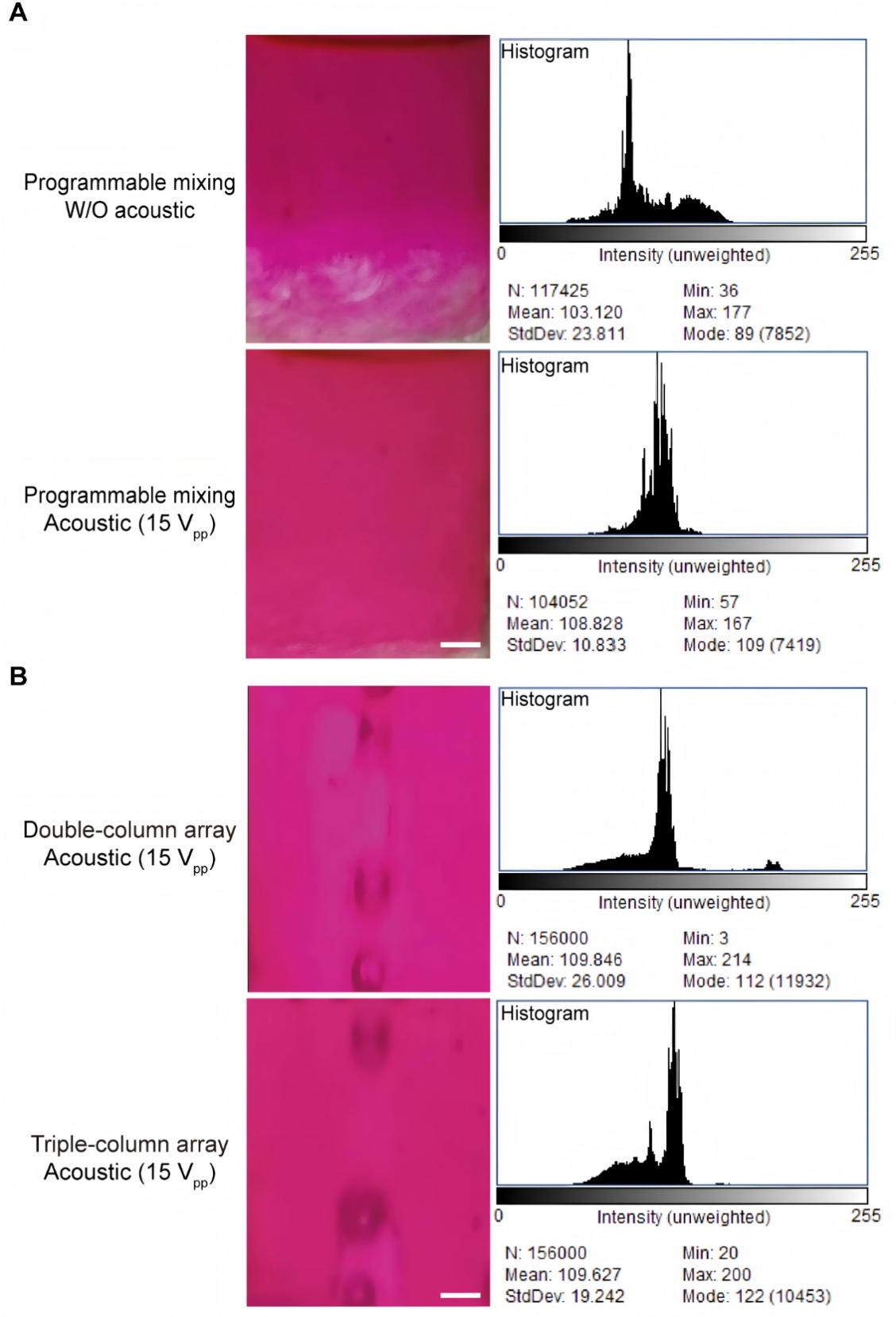


**Fig. S14. Histograms of mixing results calculated by ImageJ. The mixing index is quantitatively evaluated using a histogram in grayscale. The calculation utilizes the maximum and minimum values, the standardized variance, and the mean value for a full assessment of the mixing results. Generally, superior mixing is characterized by a smaller difference between the minimum and maximum values and a lower standard deviation. Scale bar: 500**$\text{ μm}$**.**


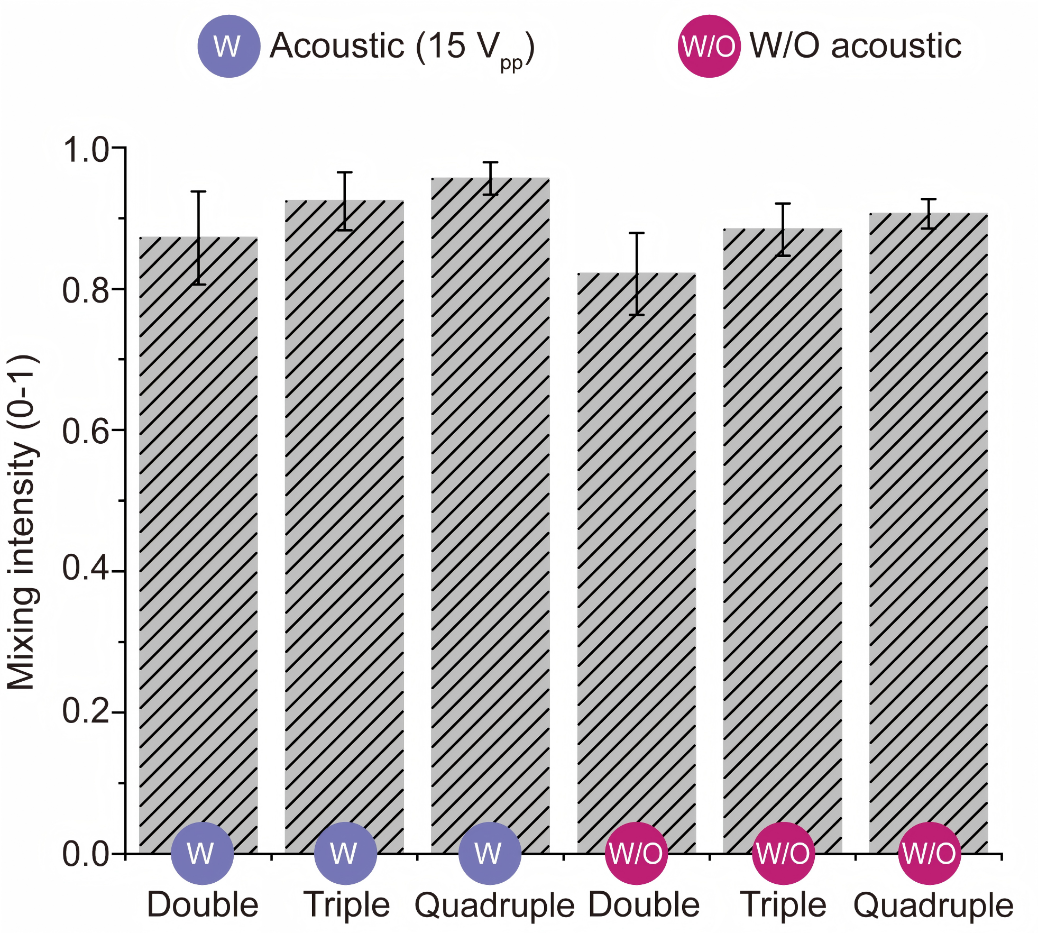


**Fig. S15. The final mixing indices of rising bubble arrays (two, three, and four columns) with or without acoustic waves during the mixing of high-viscosity materials (glycerol-to-water ratio of 1:1). This figure presents quantitative data on the mixing index, proving that acoustic bubble oscillation significantly improves the mixing index during the 8-second mixing process, and that increasing the number of bubble columns further promotes more thorough mixing.**


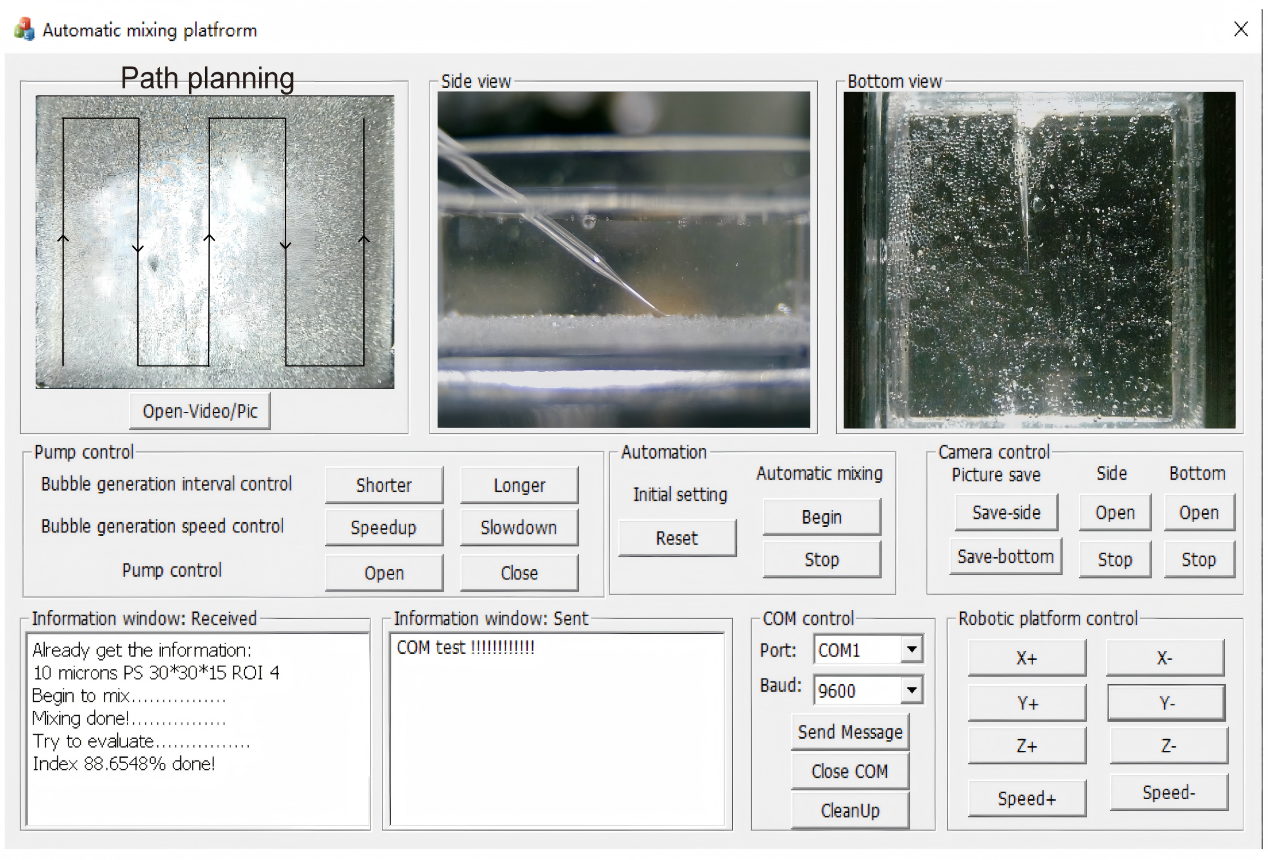


**Fig. S16. QT interface for controlling mixing operations and output of results. This figure illustrates the control interface for the programmable mixing system. It shows that the bubble generation speed and interval can be regulated to meet different mixing requirements, and that programmable mixing, assisted by path planning, enables complete mixing. Following the mixing completion, the mixing index is calculated using images from the side and bottom views.**


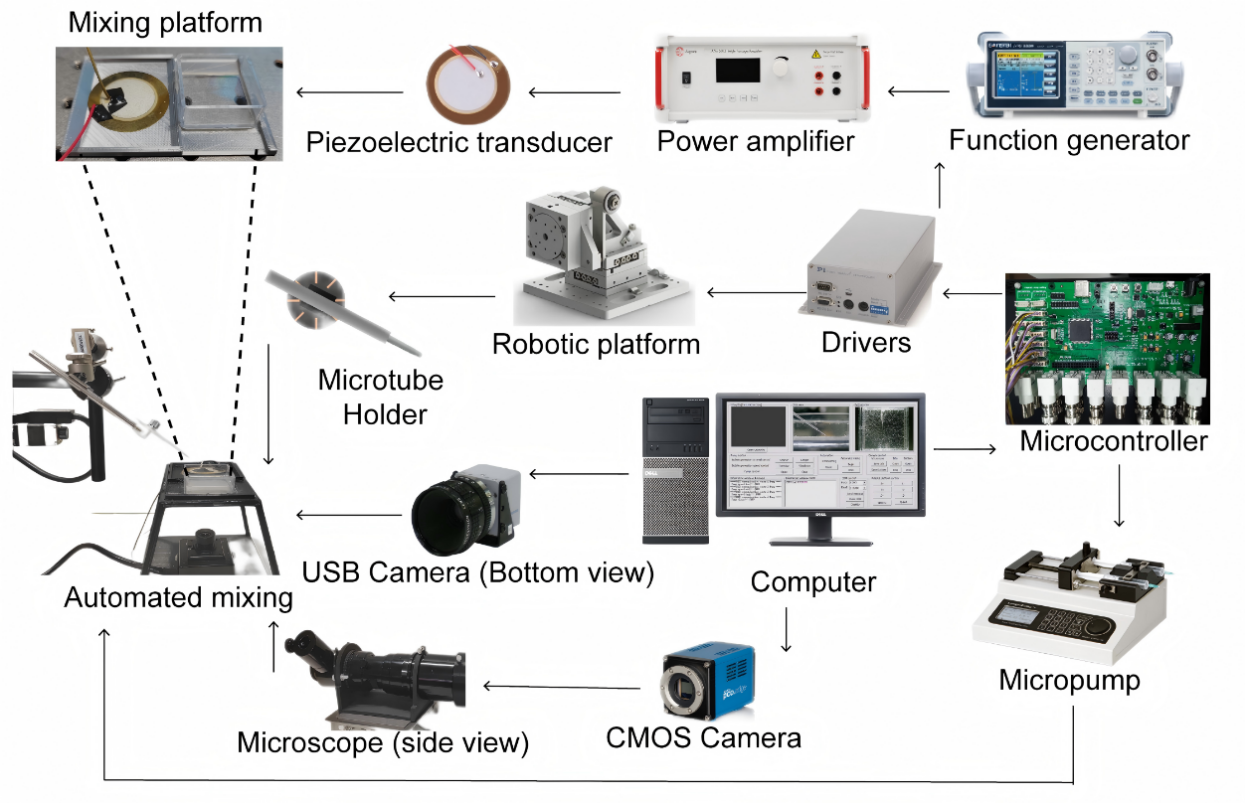


**Fig. S17. Configuration of the programmable mixing system. Schematic diagram illustrating the communication and control architecture of the system. The syringe pump, function generator, and X-Y-Z robotics platform communicate with the computer via a microcontroller to control the entire mixing process. Images captured by the orthogonal lenses are transmitted directly back to the computer through the camera's USB connection for real-time calculation of the mixing index.**


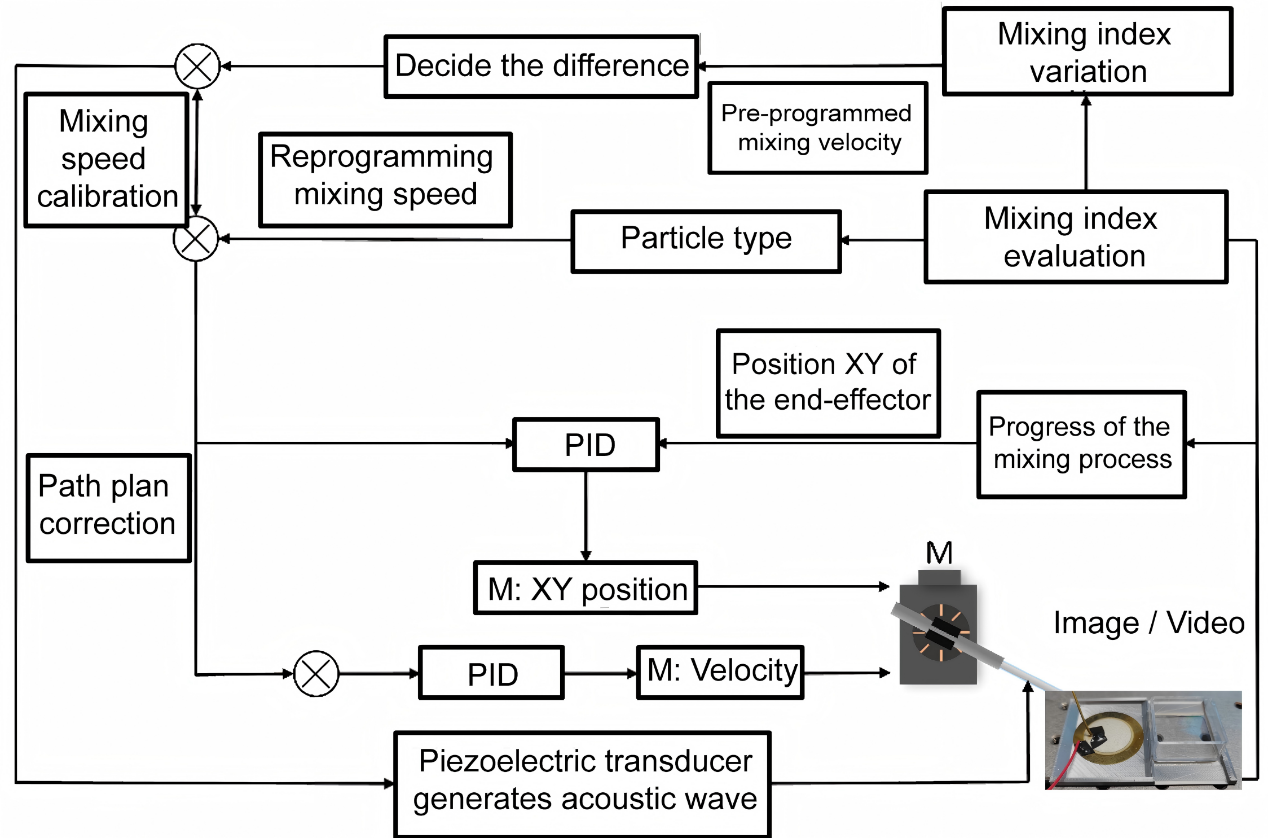


**Fig. S18. Feedback control based on the mixing index to achieve adequate mixing. This flow chart illustrates how the system calculates the difference between the current mixing index and a preset target value, and uses this difference to further control the bubble's speed and position, thereby modifying the entire path planning to achieve complete mixing.**


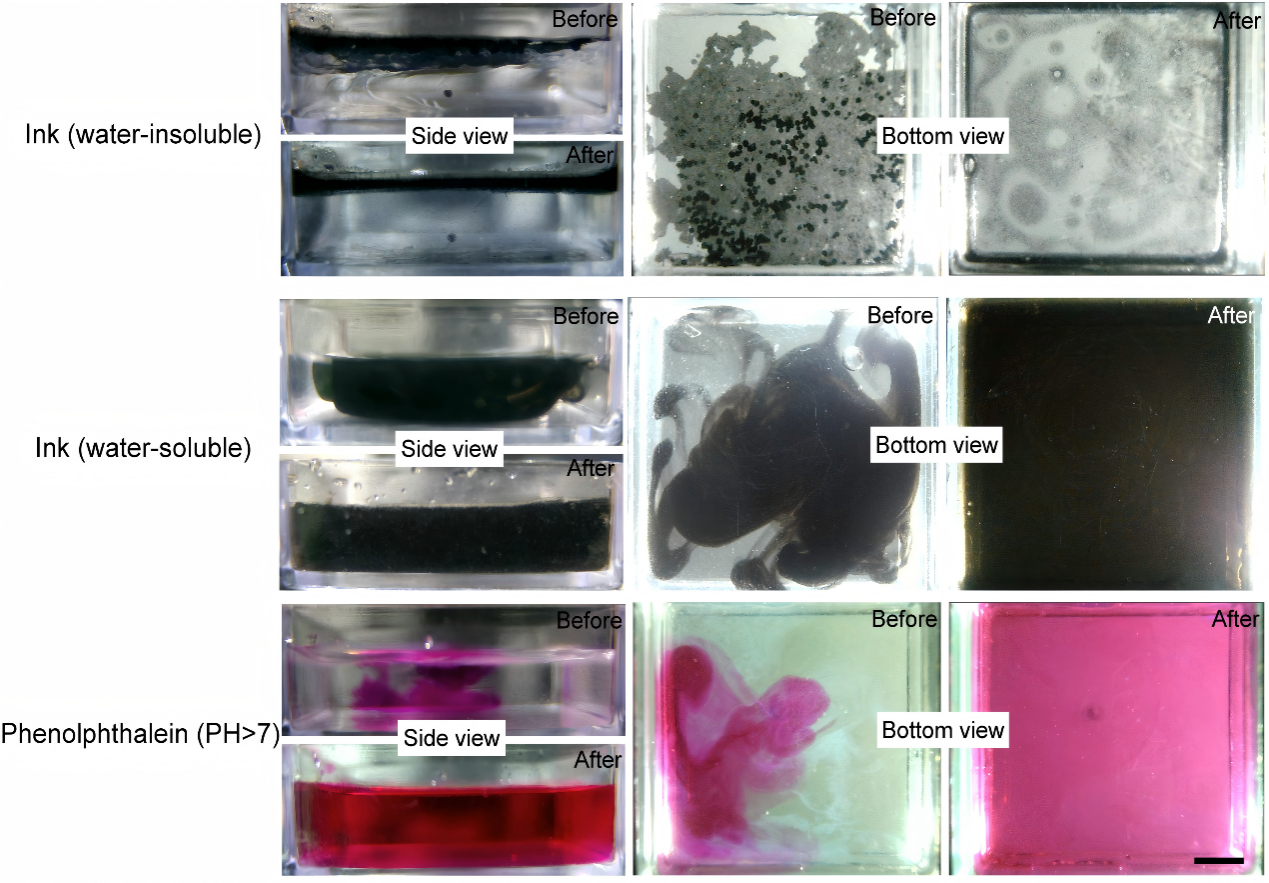


**Fig. S19. Results of programmable mixing system for chemical material synthesis. Side-view and bottom-view images showing the before-and-after results of mixing oil-based ink, water-based ink, and alkaline solution in water. The device achieves thorough mixing within 2 minutes and successfully handles relatively large liquid volumes with excellent mass transfer efficiency. Scale bar: 4**$\mathrm{mm}$**.**


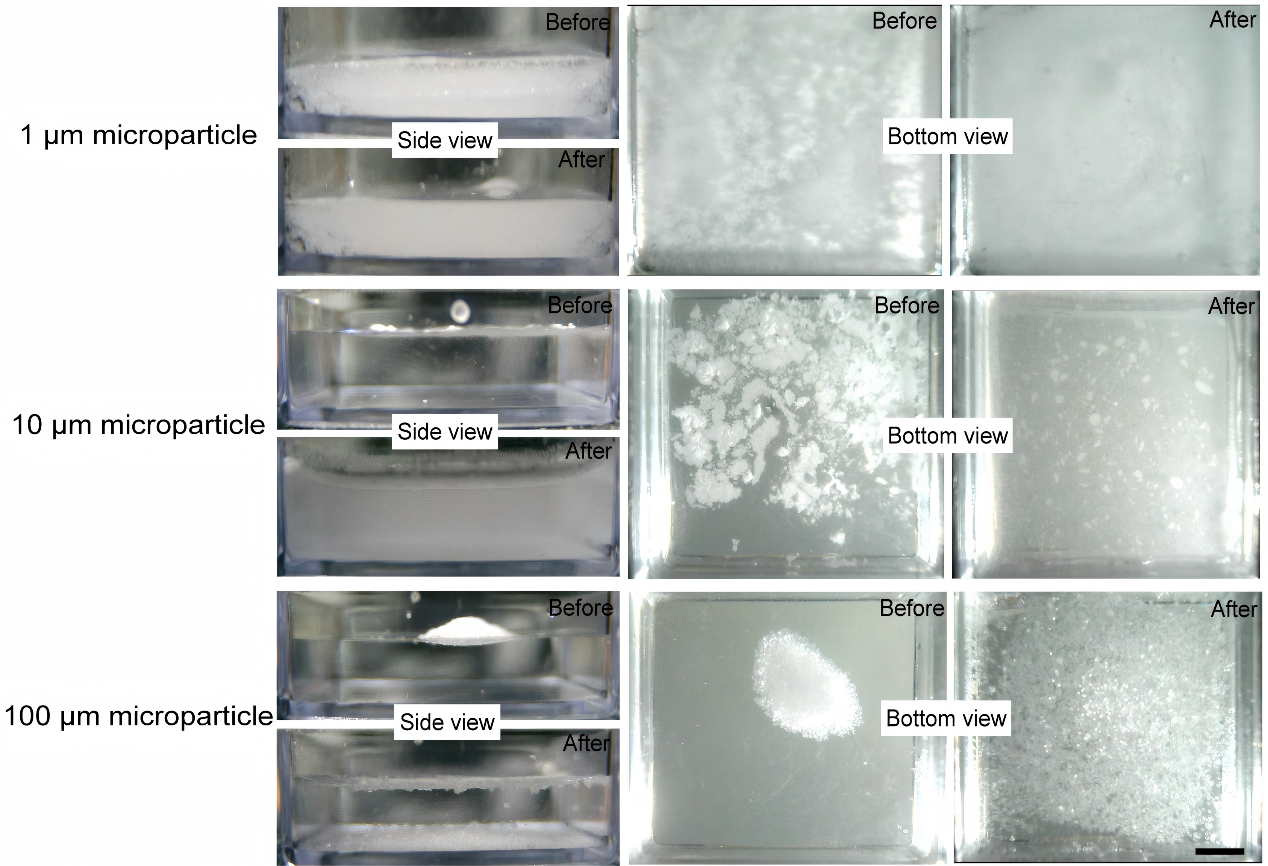


**Fig. S20. Results of programmable mixing system for particle dispersion. Side-view and bottom-view images showing the before-and-after dispersion of 1**$\text{ μm}$**, 50**$\text{ μm}$**, and 100** $\text{μm}$ **diameter PS particles in water. The device achieves complete particle dispersion within 4 minutes, confirming that acoustic rising bubbles disrupt laminar boundaries and promote the homogeneous distribution of microtargets in water. Scale bar: 4**$\text{ mm}$**.**

Movie S1. 2D microstreaming by trapped bubble oscillation at various frequencies and voltages. At the resonance frequency of 6.2kHz, the maximum range of mixing can be realized, and the effect of voltage on the mixing effect increases from 10V_pp_ to 20V_pp_.

Movie S2. 3D microstreaming by suspended bubble oscillation with various voltages at the response frequency. The mixing region is approximately a sphere, and the increase in voltage reflects an increase in the mixing effect.

Movie S3. Acoustic oscillation during bubble rising and streamlines of acoustic rising bubbles with various parameters. The oscillation of the bubbles during the rise was captured under a high-speed camera (6000 FPS), and other experiments show the effect of frequency on acoustic rising bubbles.

Movie S4. Simulation of large-scale buoyancy-driven flow and localized acoustic microstreaming. The velocity of the rising bubble affects the effect of the buoyancy-driven flow, while the simulation also shows the mixing effect of the acoustic rising bubble at different frequencies.

Movie S5. Acoustic rising bubble arrays for efficient mixing of high-viscosity fluids. Acoustic rising bubble array enhances mixing efficiency as the number of bubble columns increases when processing high-viscosity fluids.

Movie S6. Acoustic rising bubbles for accelerating chemical reaction and thrombus clearance. Experiments demonstrated the effect of acoustic rising bubbles in the reaction of calcium hydroxide with carbon dioxide to produce precipitation, in saponification reactions, and thrombus clearance.
